# Spatial Profiling Reveals Distinct Molecular and Immune Evolution of Mouse Lung Adenocarcinoma Precancers with or Without Carcinogen Exposure

**DOI:** 10.1002/advs.202512597

**Published:** 2026-01-25

**Authors:** Bo Zhu, Muhammad Aminu, Pingjun Chen, Jian‐Rong Li, Chuanpeng Dong, Chenyang Li, Yanhua Tian, Shao‐Wei Lu, Hong Chen, Chenxi Ma, Xin Hu, Jie Ye, Andrew Y. Liu, Beibei Huang, Frank R. Rojas, Parra Cuentas. Edwin Roger, Ou Shi, Monique B. Nilsson, Alissa Poteete, Khaja B. Khan, Wei Lu, Luisa M. Solis Soto, Junya Fujimoto, Cara Haymaker, Ignacio I. Wistuba, Zhubo Wei, Linghua Wang, Don L. Gibbons, Ken Chen, Alexandre Reuben, Jason M. Schenkel, John V. Heymach, Chao Cheng, Jia Wu, Jianjun Zhang

**Affiliations:** ^1^ Departments of Thoracic/Head and Neck Medical Oncology The University of Texas MD Anderson Cancer Center Houston Texas USA; ^2^ Genomic Medicine The University of Texas MD Anderson Cancer Center Houston Texas USA; ^3^ Imaging Physics The University of Texas MD Anderson Cancer Center Houston Texas USA; ^4^ Institute for Data Science in Oncology The University of Texas MD Anderson Cancer Center Houston Texas USA; ^5^ Translational Molecular Pathology The University of Texas MD Anderson Cancer Center Houston Texas USA; ^6^ Department of Medicine Baylor College of Medicine Houston Texas USA; ^7^ Department of Genetics Yale University School of Medicine New Haven Connecticut USA; ^8^ Department of Cancer Systems Imaging The University of Texas MD Anderson Cancer Center Houston Texas USA; ^9^ Clinical Research Center in Hiroshima Hiroshima University Hospital Hiroshima Japan; ^10^ Department of Bioinformatics & Comp Biology The University of Texas MD Anderson Cancer Center Houston Texas USA

**Keywords:** imaging mass cytometry, spatial single cell, spatial transcriptomics, whole‐exome sequencing

## Abstract

Tumor evolution involves genetic, transcriptional, and phenotypic alterations that shape cancer cell behavior and interactions with the microenvironment. While single‐cell technologies have advanced our understanding of this process, spatial dynamics remain incompletely characterized. Here, whole‐exome sequencing (WES), imaging mass cytometry (IMC), and spatial transcriptomics (ST) were integrated to study molecular evolution and immune responses in two lung adenocarcinoma (LUAD) mouse models: a genetically engineered model (129S4/Sv‐Kras^LSL‐G12D^, termed 129S4 K) and a carcinogen‐induced precancer model (129S4 U). Compared to 129S4 K, 129S4 U tumors exhibited higher mutational, neoantigen but lower copy number variation (CNV) burdens at matched developmental timepoints, consistent with findings of higher mutational burden in human smoking‐related LUAD than nonsmoking LUAD. We profiled over 1.4 million spatial single cells from 284 IMC regions of interest and 51,531 spatial transcriptomic spots from 156 lesions across 141 mice. Macrophage abundance increased with tumor progression, while CD8 T‐cell and B‐cell densities declined in late‐stage LUAD. 129S4 U showed greater immune infiltration in both tumor and adjacent normal tissue, higher T‐cell cytotoxicity signature score in line with its higher mutational and neoantigen burdens. LUAD progression was marked by early morphological shifts and late‐stage changes in cell states and interactions. These data define spatial and genetic landscapes of LUAD development and provide a framework for investigating immune evolution and therapeutic strategies in early carcinogenesis.

## Graphical abstract

Tumor evolution in lung adenocarcinoma is shaped by genetic alterations and spatial immune dynamics. By integrating whole‐exome sequencing, imaging mass cytometry, and spatial transcriptomics across two mouse models, this study reveals how mutational burden, immune infiltration, and cell–state interactions evolve during early and late carcinogenesis.

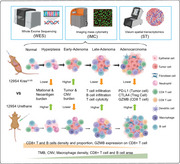

## 1 Introduction

Despite advances in both treatment and early diagnosis, lung cancer remains the leading cause of cancer associated death globally [1]. Lung adenocarcinoma (LUAD) is the most common histological subtype of lung cancer, accounting for approximately roughly 50%–60% of non‐small cell lung cancer (NSCLC) cases [2]. Atypical adenomatous hyperplasia (AAH) is the only recognized LUAD precancer with potential to progress to LUAD precursors including preinvasive adenocarcinoma in situ (AIS), minimally invasive adenocarcinoma (MIA), and ultimately invasive LUAD (IAC) [3]. Radiologically, AAH, AIS, MIA, and some IAC present as ground‐glass opacity or subsolid pulmonary nodules [4, 5, 6]. Moreover, these LUAD precursors have become increasingly common due to the widespread implementation of lung cancer screening and the advent of high‐resolution computed tomography [7]. While some pulmonary nodules remain stable for years, others progress to become IAC. Despite the increased detection rate, the factors that dictate nodule stability versus progression remains unclear, and a better understanding of tumor intrinsic and extrinsic mechanisms that mediate progression is sorely needed. Detecting and treating LUAD in premalignant or preinvasive stages, termed interception, holds significant promise for improving lung cancer associated mortality [8]. A clear understanding of how tumors and the tumor microenvironment (TME) change remains critical for identifying pathways and cell types that can be perturbed for interception.

Over the past decade, sequencing‐based approaches have been widely employed to both mechanistically understand tumor evolution and to identify therapeutic vulnerabilities that can be harnessed clinically [9, 10, 11, 12, 13]. Recent studies employing whole‐exome sequencing (WES) or whole‐genome sequencing (WGS) on clinical tissue samples have unveiled genomic evolution at the single nucleotide level, delineated chromosomal evolution, and identified epigenetic aberrations [14, 15]. Single‐cell RNA sequencing (scRNA‐seq) on freshly resected lung specimens has pinpointed AT2‐like cells, club cells, or basal cells as the main LUAD progenitor cells [13, 16]. While these studies underscore the pivotal role of genomic characterizations and transcriptional features across LUAD evolution, they have yet to elucidate how the spatial TME changes during tumor progression and evolution. Thus, it will be critical to identify the relationship between tumor and immune interactions in the spatial context to predict whether precancers will regress, remain stable, or progress.

While human studies are essential for identifying key pathophysiological aspects of tumor evolution, there are considerable hurdles that remain preventing a more mechanistic understanding of LUAD. For example, human specimens have several inherent limitations for LUAD precancer evolution studies including (1) surgical resection is not the standard of care for managing LUAD precursors, resulting in a scarcity of resected LUAD precursor samples; (2) a large number of samples are likely needed to control for and observe the profound inter‐patient heterogeneity; and (3) interventional studies can take a long time to accrue and analyze relevant samples. On the other hand, genetically engineered mouse models (GEMMs) and carcinogen‐induced models are excellent model systems, which enable mechanistic studies into the underlying cancer biology. These models closely mimic human malignancies with a relevant immune infiltrate. Moreover, the controlled genetic background, lifestyle, and homogeneous biological features make them ideal for studying cancer evolution longitudinally. Multiple murine LUAD models have been generated including the genetically engineered mouse model of lung cancer (e.g., 129S4/Sv‐Kras^LSL‐G12D^; 129S4 K) and carcinogen‐induced model of lung cancer (e.g., 129S4/Sv urethane induced; 129S4 U) [17, 18, 19, 20, 21]. scRNA‐seq, cytometry by time of flight (CyTOF), and fluorescence‐activated cell sorting (FACS) have offered insights into the molecular mechanisms driving tumorigenesis and immunosurveillance in these mouse models [22]. However, sample processing for these methods (e.g., tissue dissociation) obliterates tumor spatial information and prevents insight into cell–cell interactions.

Here, we employed multiplexed tissue imaging mass cytometry (IMC) and 10× Visium spatial transcriptomics (ST) to identify how TME spatial characteristics differ in LUAD precursors from GEMM LUAD versus carcinogen‐induced LUAD models. We profiled diverse immune and epithelial cell populations throughout different stages of LUAD development, examining their cellular compositions and interactions, spatial developmental trajectories, and transcriptional states. Our comprehensive study offers detailed insight into the relationship between molecular evolution and immune/epithelial spatial alterations during mouse LUAD progression. Moreover, we reveal distinct TME profiles between 129S4 K and 129S4 U as tumors develop, providing valuable resources for future studies and therapeutic approaches.

## 2 Results

### 2.1 Distinct Genomic Features in Carcinogen Versus Genetic LUAD Models

**FIGURE 1:**
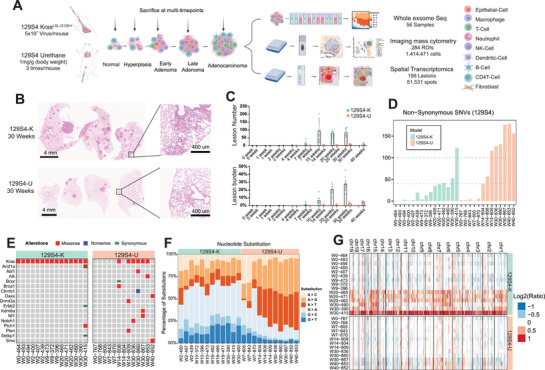
Whole‐exome sequencing reveals the lesion mutation burden of 129S4 Kras^G12D^ and 129S4 urethane. (A) Schematic showing the precancer mouse models’ establishment and multimodal data analysis. Images were created with BioRender. (B) Representative H&E staining images of 30 weeks lung tissue of 129S4 K and 129S4 U models. Scale bars, 4 mm or 400 µm. (C) Lesion number and burden comparisons of 129S4 Kras^G12D^ and 129S4 urethane models at matched timepoint (*p* < 0.05, two‐tailed Student's test). (D) Nonsynonymous SNVs (Single Nucleotide Variant) per lesion, light green represents the 129S4 Kras^G12D^ model and light red represents the 129S4 urethane model. All comparisons of Nonsynonymous SNVs between two groups at similar timepoint were significant (*p* < 0.05, two‐tailed student's test). (E) All missense (Mis), nonsense (Non) SNVs, synonymous in genes listed are displayed. (F) Trinucleotide transition across different timepoints samples from 129S4 K and 129S4 U models. (G) Distinct copy number profiles of 129S4 Kras^G12D^ and 129S4 urethane‐induced lesions.

To induce lung tumors, 129S4/Sv‐^Kras4Tyj/J^ mice (129S4 K model; Figure 1A) were given adenovirus intranasally into the lungs or wild‐type 129S4/Sv mice were injected with urethane into the peritoneal cavity [23, 24] (129S4 U model; Figure 1A). Afterwards, mice were sacrificed at defined timepoints, and lung tissue samples were formalin‐fixed, and embedded in paraffin. Hematoxylin and eosin (H&E) staining revealed hyperplasia 4 weeks after tumor initiation in both models (Figure S1A,B). Notably, 129S4 K exhibited a significantly higher number and size of neoplastic lesions than 129S4 U across timepoints during tumor development (Figure 1B,C).

Analysis of WES data demonstrated progressive increase of total mutation burden (TMB) from early to later stages, consistent with human LUAD precancer data [14]. Compared to 129S4 K, 129S4 U had a significantly higher number of nonsynonymous mutations and oncogene mutations suggesting greater mutation rates in the carcinogen‐induced model compared with the genetic 129S4 K model (Figure 1D,E and Figure S1C). Notably, in addition to Kras G12D in the K model and Kras Q61H in the U model, mutations were identified in several genes frequently altered in human LUAD, including NF1, NOTCH1, and CDH11. These findings indicate that the molecular landscapes of both models closely recapitulate key features of human LUAD (Figure S1C). On the other hand, the copy number variation (CNV) burden was higher in the 129S4 K than the 129S4 U model. These results are consistent with previous reports examining genomic alterations in these models [17, 25] indicating different genomic alternations associated with carcinogenesis of genetically engineered versus carcinogen‐induced LUAD models.

Consistent with overall mutation burden, the predicted neoantigen burden was also higher in the 129S4 U model than the 129S4 K model (Figure S1D). We identified 492 genes associated with predicted neoantigens in the 129S4 U model, compared with 280 genes in the 129S4 K model. Leveraging spatial transcriptomics data, analysis of the 11 neoantigen‐associated genes shared between the two models revealed consistently higher expression of these genes in the 129S4 U model (Figure S1E), supporting a greater neoantigen burden and potentially enhanced immunogenicity in this carcinogen‐induced LUAD model. Importantly, these findings are concordant with observations in human LUAD, where smoking‐associated tumors exhibit higher mutational and neoantigen burdens than nonsmoking LUAD in The Cancer Genome Atlas Program (TCGA) cohort (Figure S2A–D). Because both 129S4 K and 129S4 U models harbor Kras mutations (Figure 1E), we further focused our analysis on KRAS‐mutant LUAD in TCGA stratified by smoking status, which similarly revealed significantly higher mutation and neoantigen burdens in tumors from smokers (Figure S2E–G).

Mutation spectrum analysis revealed increased A > G and A > T transitions with neoplastic progression in 129S4 U, while 129S4 K demonstrated predominately G > A across different timepoints (Figure 1F). We further examined the 96 substitution patterns used to derive The Catelogue Of Somatic Mutations In Cancer (COSMIC) mutational signatures [26] in the 129S4 U model to elucidate the mechanisms underlying urethane‐induced mutagenesis. The results demonstrated that A > G and A > T groups are the most frequent substitutions in 129S4 U (Figure S2H), consistent with previously published data from urethane‐induced LUAD models [17, 27]. It is important to note that the predominant substitutions from human LUAD of smokers are G > A and G > T. These results underscore the similarity (G > A transitions) as well as the differences between human tumors (Figure S2I) and mouse models (A > G in mouse models vs. G > T in human LUAD tumors), reflecting differences in genomic backgrounds (between human and mice) and the carcinogen exposures—cigarette smoking producing more than 7000 chemicals including at least 70 known carcinogens versus pure urethane [28]—impacting the mutation patterns during carcinogenesis.

Next, we leveraged spatial transcriptomics data to investigate the functional impact of the elevated CNV burden observed in the 129S4 K model. We first defined CNV‐high and CNV‐low tumor regions (Figure S2J). Analysis of the distribution of oncogenes and tumor suppressor genes within CNV‐high regions revealed preferential deletion of tumor suppressor genes and amplification of oncogenes (Figure S2K). We next examined the expression of canonical tumor suppressor genes and oncogenes in CNV‐high versus CNV‐low regions. Compared with CNV‐low regions, CNV‐high regions exhibited significantly reduced expression of multiple tumor suppressor genes, including Cdkn2a, Rb1, Pten, and Tsc1 (Figure S2L), alongside significantly increased expression of oncogenes such as Braf, Egfr, and Erbb2 (Figure S2M). Together, these findings underscore the functional relevance of CNV alterations in driving oncogenic programs in this model.

Furthermore, we performed counterfactual comparisons to determine whether differences in CNV burden are merely a byproduct of differences in TMB, and vice versa. Specifically, we compared CNV burden between 129S4 K and 129S4 U models within TMB‐matched strata and compared TMB levels between these two models within CNV‐matched strata. Within low, intermediate, and high TMB strata, 129S4 K tumors consistently exhibited numerically higher CNV burden than 129S4 U tumors (Figure S2N). Conversely, within CNV‐matched strata, 129S4 U tumors consistently maintained higher TMB compared with 129S4 K tumors (Figure S2O). Although many of these differences did not reach statistical significance—likely due to limited sample size—together, these observations highlight distinct patterns of genomic alteration underlying tumor development in carcinogen‐induced versus genetically engineered models.

### 2.2 IMC Profiling Spatial Single Cell Landscape of Carcinogen and Genetic LUAD Models during Tumor Evolution

**FIGURE 2:**
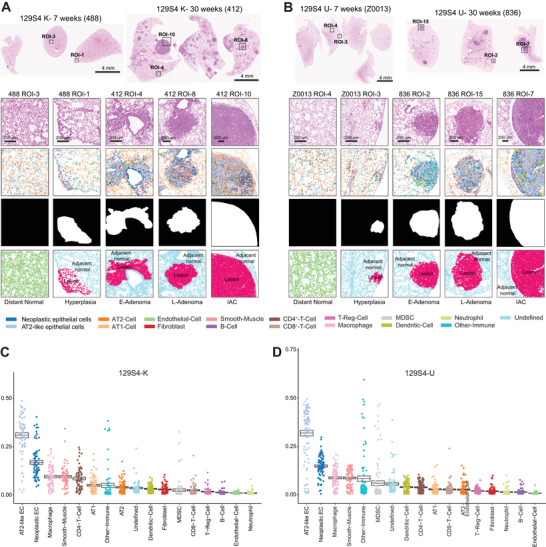
Imaging mass cytometry reveals the immune evolution of 129S4 K and 129S4 U models. (A,B) Top: Representative H&E image showing the mouse whole lung tissue from different sacrifice timepoints of 129S4 K and 129S4 U models, Scale bars, 4 mm. First line: Zoom in H&E staining images showing the different histological tissue of 129S4 K and 129S4 U models. Scale bars, 200 µm. Second line: Representative image of different pathological lesions showing the spatial localization of 17 major cell types, each color refers to the bottom represents one cell type. Third line: Representative image of segmentation mask showing the lesion area and non‐lesion area by different colors, black (non‐lesion area), white (lesion area). Bottom: Highlighted regions of distant normal, lesion adjacent normal and lesion area by different colors, light green (distance normal), light blue (lesion adjacent normal), and light red (lesion). (C,D) Prevalence of 17 cell types as a proportion of total cells across different pathological lesions of 129S4 K and 129S4 U models.

To examine how TME changes over the course of LUAD carcinogenesis, we applied IMC using a 39‐plex antibody panel [23] (Table S1 and Figure S3A), mouse lungs collected at different timepoints (Figure S3B). Using unsupervised clustering [23] (Table S2), we identified a total of 1,414,471 single cells that were classified into 17 cell types of 4 major categories: “epithelial” (neoplastic epithelial, AT2‐like neoplastic epithelial, AT1‐like epithelial cells, and AT2‐like epithelial cells), “lymphoid” (comprising T and B cells), “myeloid” (macrophages, Myeloid‐Derived Suppressor Cells (MDSC), dendritic cells (DCs), and neutrophils), and “stromal” (endothelial, fibroblast, and smooth muscle cells). Multiple Region of Interest (ROIs) were selected based on different histological stages (Figures S4A and S5A, and Table S3) including normal lung, hyperplasia (resembling human AAH), early adenoma (resembling human AIS), late adenoma (resembling human MIA), and adenocarcinoma (resembling human IAC) (Figure 2A,B). Notably, normal lung tissues and lesion adjacent normal were predominantly comprised of AT1‐like and AT2‐like epithelial cells, while the lesion area exhibited a higher prevalence of neoplastic epithelial and AT2‐like neoplastic epithelial cells (Figure 2A,B).

### 2.3 Greater Immune Infiltration in the Carcinogen‐Induced LUAD Model than the Genetic LUAD Model

**FIGURE 3:**
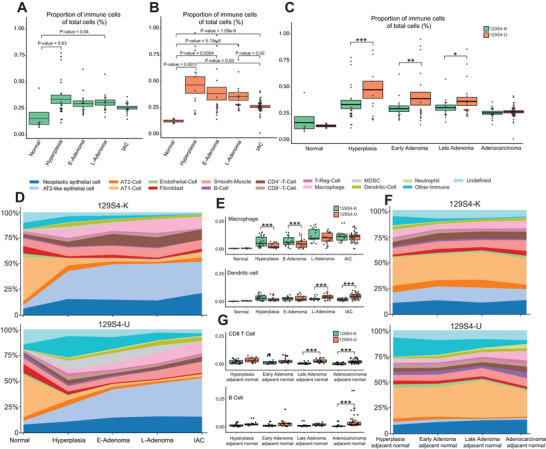
Spatial single‐Cell Landscape of Immune, epithelial, and Stromal Cells at Different Stages of mouse LUAD of 129S4 K and 129S4 U models. (A,B) Immune cells proportion of total cells in lesion area across different pathological lesions of 129S4 K (green color) and 129S4 U (orange color) model. (Pairwise multiple Welch's *t*‐tests with Bonferroni correction). (C) Comparison of proportion of immune cells as total cells in lesion area across different pathological lesions between 129S4 K (green color) and 129S4 U (orange color) models. (* *p* < 0.05, ** *p* < 0.01, and *** *p* < 0.001, two‐tailed Student's test). (D) Compositions of total TME cells of 129S4 K (upper panels) and total TME cells of 129S4 U (lower panels) across pathological lesions. Cells from the normal and lesion area as shown in Figure 2A,B. (E) Representative figures showing the comparison of prevalence of individual cell types across different pathological lesions between 129S4 Kras^G12D^ and 129S4 urethane models’ lesion area (* *p* < 0.05, ** *p* < 0.01, and *** *p* < 0.001, two‐tailed Student's test). (F) Compositions of total TME cells of 129S4 Kras^G12D^ (upper panels) and total TME cells of 129S4 urethane (lower panels) across pathological lesions. Cells from the lesion adjacent normal area as shown in Figure 2A,B. (G) Representative figures showing the comparison of prevalence of individual cell types between 129S4 K and 129S4 U models’ lesion adjacent normal area. (* *p* < 0.05, ** *p* < 0.01, and *** *p* < 0.001, two‐tailed Student's test).

Analysis of the TME within LUAD and precursor lesions (highlighted in red in Figure 2A,B) revealed that AT2‐like neoplastic epithelial cells were the predominant cells in both 129S4 K (30.4% of total cells) and 129S4 U (31.5% of total cells) models, while macrophages were the dominant immune cells in both models (Figure 2C,D). Compared to normal lung tissues, immune cell density was higher in LUAD and its precursors in both 129S4 K and 129S4 U (Figure S6A,B) indicating an ongoing immune response accompanying tumor development and progression. However, the proportion of immune cells peaked early at hyperplasia stage, and then subsequently decreased as tumors continued to evolve (Figure 3A,B). This is consistent with our previous findings in human LUAD and its precursors [29] suggesting progressive immune exclusion as LUAD precancers progress over time. Additionally, the immune cell density was significantly higher in 129S4 U lesions as compared to 129S4 K lesions across all stages from hyperplasia to IAC (Figure S6C). The proportion of immune cells of total cells was also significantly higher in 129S4 U than 129S4 K at hyperplasia, early adenoma, and late adenoma stages, which became similar at the adenocarcinoma stage (Figure 3C). These findings align with the higher TMB observed in 129S4 U mice relative to 129S4 K mice, which could result in increased neoantigen load and a more robust immune response. These observations are consistent in those in human lung cancers that tumors with high TMB exhibiting significantly greater immune infiltration than TMB‐low tumors [30]. It is also worth noting that analysis of KRAS‐mutant LUAD tumors from the TCGA cohort also demonstrated higher TMB and tumor‐infiltrating lymphocyte levels in smokers compared with nonsmokers (Figure S2G) highlighting the impact of carcinogens on genomic and immune landscape of LUAD.

Next, we examined how major immune subsets changed during early LUAD evolution. In both 129S4 U and 129S4 K models, macrophages and CD4^+^ T cells progressively increased, while B cells progressively decreased from normal to hyperplasia, E‐Adenoma, L‐Adenoma, and IAC (Figure S6D,E). Moreover, CD8^+^ T cells were increased in early‐stage lesions compared to normal tissue. However, this was followed by CD8^+^ T‐cell contraction as tumors progressed into later‐stage disease (Figure S6D,E). These results are consistent with human LUAD precursor data [29], indicating a dynamic immune activation and immune evasion with LUAD precancer evolution in both models with what appears to be an overall more active immune response at precancer stages and an ultimate decrease of antitumor immune response in invasive LUAD. On the other hand, 129S4 K and 129S4 U demonstrated different regulatory T‐cell (Treg) and DC infiltration patterns over the course of tumor progression (Figure S6F). In 129S4 U, Tregs and DCs progressively increased with neoplastic evolution. However, in 129S4 K, Tregs and DCs were increased in early‐stage disease followed by a concomitant decrease in later stages. In line with the overall immune cell density data, the cell density of CD8^+^ T cells, DCs, B cells, and Tregs was significantly higher in 129S4 U than 129S4 K across different stages, particularly in the later disease stages (Figure S6D–F), suggesting that there is an overall more robust immune response in 129S4 U than 129S4 K. However, 129S4 K had significantly higher macrophage proportions at hyperplasia, E‐Adenoma stages (Figure 3D,E) and significantly higher CD4^+^ T‐cell proportions at E‐Adenoma, L‐Adenoma, and IAC stages than 129S4 U (Figure S6G–I). Taken together, these results suggested different trajectories of immune response during early LUAD carcinogenesis between carcinogen‐induced versus genetic models.

To further define the timing of the transition from early immune activation to later immune suppression, we leveraged IMC data integrated with pathologic staging annotated by expert thoracic pathologists to assess immune dynamics across disease stages. We examined granzyme B (GZMB) expression in CD8^+^ T cells across stages. GZMB expression initially increased during the hyperplasia and early adenoma stages, followed by a marked decline after the late adenoma stage in both models (Figure S6J,K). Collectively, these results identify a critical transition during early carcinogenesis—specifically from hyperplasia to early adenoma—marked by a shift from broad immune activation to immune suppression and tighter immune regulation. Notably, this transition represents a shared immunologic trajectory across both carcinogen‐induced and genetically engineered Kras‐mutant LUAD precancer models.

The immune microenvironment of tumor adjacent lung tissues can provide critical information regarding host antitumor immune response and patient survival [31]. To understand the immune microenvironment surrounding these LUAD precursor lesions, we next examined the immune cell infiltration at the lesion adjacent normal lung area during LUAD evolution (Figures 2A,B and 3F,G, and Figure S7A–L). In 129S4 K, the overall immune proportion, including CD8^+^ T cells, B cells, Tregs and DCs, (Figure S7A) and immune density (Figure S7G) decreased in later stages (Figure S7D). However, the infiltration of macrophages and CD4^+^ T cells increased in later stages (Figure S7D). The lesion adjacent normal of 129S4 U had a similar trend in the overall immune cell infiltration (Figure S7B,H) and most immune subsets (Figure S7E,K). However, the infiltration of Tregs and DCs was significantly higher at later stage lesions of the 129S4 U model, the opposite of 129S4 K (Figure S7F,L). Comparing the immune distribution between the two models, the immune cell proportion of total cells was higher in 129S4 U than 129S4 K at all stages of the lesion adjacent normal (Figure S7C), and immune cell density was higher at E‐Adenoma and IAC lesion adjacent normal (Figure S7I). Additional assessments revealed higher CD8^+^ T‐cell and B‐cell proportions of total cells (Figure 3F,G), as well as higher cell density of CD8^+^ T cells, B cells, Tregs, and dendritic cells in 129S4 U than 129S4 K at the late‐stage lesion adjacent normal (Figure S7L), indicating an overall more robust antitumor immune microenvironment in the lesion surroundings in carcinogen‐induced models.

### 2.4 Stronger Functional States and Tighter Regulation of T Cells in the Carcinogen‐Induced LUAD Model than the Genetic Model

Beyond immune cell infiltration, immune functional states play a critical role in shaping antitumor immunity. We therefore leveraged spatial transcriptomics data to infer immune cell functional states in the two LUAD models, with a particular focus on T‐cell cytotoxicity and exhaustion signatures. T‐cell cytotoxicity was assessed using established marker genes (Gzmb, Gzma, Prf1, Fasl, Ifng, Tnf, Nkg7, Cd107a, Lamp1, Ccl5, Cd69, Cxcr3, Il2rb, Tbx21, Eomes, Runx3, Prdm1, Gzmk, Ccl4, and Cd2), while T‐cell exhaustion was evaluated using canonical markers (Pdcd1, Ctla4, Lag3, Tigit, Havcr2, Cd244, Tox, Tox2, Eomes, Nr4a1, Nr4a2, Nr4a3, Batf, Id2, Id3, Cxcr5, Prdm1, Entpd1, and Cd160). As shown in Figure S8A–D, the 129S4 U model exhibited significantly higher scores for both T‐cell cytotoxicity and T‐cell exhaustion compared with the 129S4 K model, consistent with a stronger and more sustained T‐cell response in the 129S4 U tumors.

We next examined PD‐L1 expression using both IMC and Visium spatial transcriptomics (Figure S8E–H). PD‐L1 protein expression on tumor cells was significantly higher in the 129S4 U model compared with the 129S4 K model, as assessed by IMC (Figure S8E). Concordantly, Visium data revealed minimal Cd274 (PD‐L1) expression in the 129S4 K model, whereas substantially higher expression was detected in the 129S4 U model (Figure S8F). Analysis of Ctla4 expression yielded similar results (Figure S8G,H). Collectively, these findings indicate that the urethane‐induced LUAD model exhibits a more robust antitumor T‐cell response accompanied by heightened immune checkpoint engagement, reflecting tighter regulation of T‐cell activity compared with the carcinogen‐naïve Kras model.

### 2.5 Distinct Spatial Paired Cell–Cell Interactions as Tumor Progression by Neighborhood Analysis

Next, we explored multicellular interactions in the TME during LUAD early evolution, we utilized neighborhood analysis with permutation tests [32] to quantify cellular colocalization and identify significant collaborations or avoidance patterns between cell phenotypes [33]. This allowed us to infer cell‐to‐cell interactions and communication patterns, correlating spatial relationships with histopathological features. Both 129S4 K and 129S4 U exhibited decreased interaction between AT2‐like epithelial cells and neoplastic epithelial cells as tumors progressed (Figure 2A,B; Figure S9A, box 1; and Figure S9B, box 1). Interaction between neoplastic epithelial cells and Tregs increased at later stages in both models (Figure S9A, box 2, and Figure S9B, box 2), consistent with a more immune‐suppressive TME in later stages.

**FIGURE 4:**
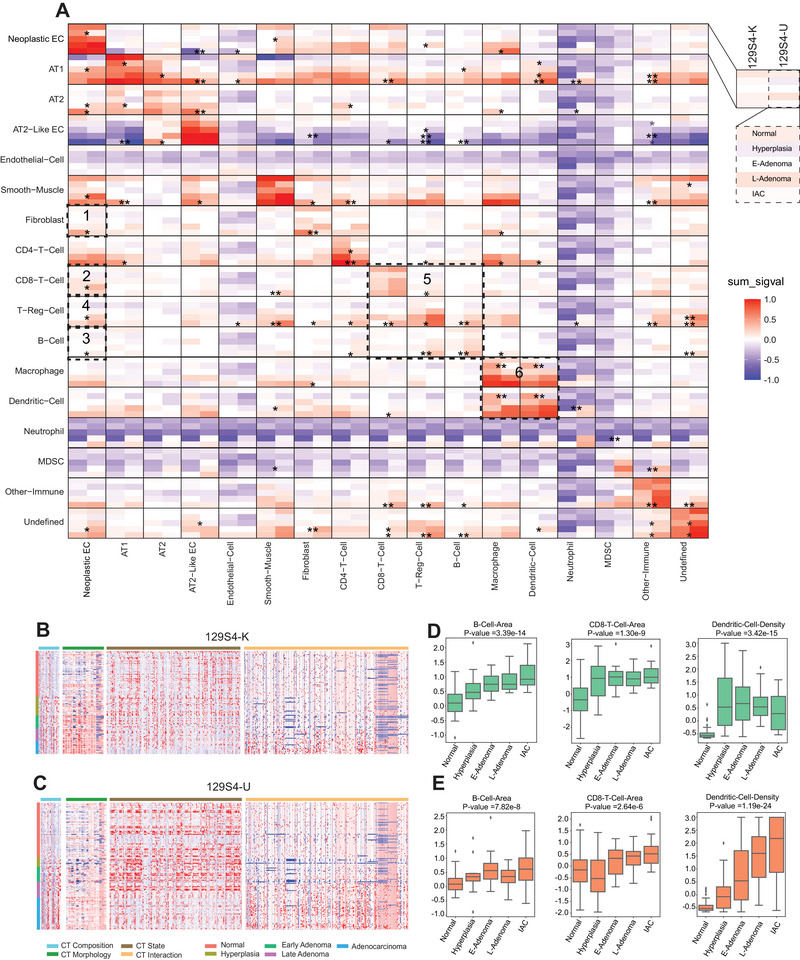
Cell–cell interaction profiles across pathological lesions in LUAD and features summary of 129S4 K and 129S4 U models. (A) Heat map depicting significant pairwise cell–cell interaction (red) or avoidance (blue) comparison between 129S4 K and 129S4 U models across the five pathological lesions (normal, hyperplasia, E‐Adenoma, L‐Adenoma, and adenocarcinoma). The black boxes depict associations referenced in the text. (* *p* < 0.05, ** *p* < 0.01, and *** *p* < 0.001, two‐tailed Student's test). (B) Features (CT composition feature = 32, CT morphology = 64, CT state = 208, and CT interaction = 256) summary of 126 ROIs across five histological stages of 129S4 K model. (C) Features (CT composition feature = 32, CT morphology = 64, CT state= 208, and CT interaction = 256) summary of 156 ROIs across five histological stages of the 129S4 U model. (D,E) Representative individual feature value across pathological lesions of (D) 129S4 K and (E) 129S4 U models. (Pairwise multiple Welch's *t*‐tests with Bonferroni correction).

Compared to 129S4 K,129S4 U displayed higher interactions of neoplastic epithelial cells with CD8^+^ T cells (Figure 4A, box 1) as well as B cells (Figure 4A, box 2). The interactions of Tregs with neoplastic epithelial cells (Figure 4A, box 3), CD8^+^ T cells (Figure 4A, box 4), and B cells (Figure 4A, box 4) are also significantly higher in 129S4 U compared to 129S4 K models, particularly at the IAC stage. These results suggested a more active and more tightly regulated immune TME in 129S4 U. Further investigation of cell–cell interactions in the lesion adjacent normal area also revealed higher interaction between CD8 T cells and neoplastic epithelial cells (Figure S9C, box 1) in 129S4 U, consistent with a more active antitumor immunity. In contrast, 129S4 K displayed higher interaction between macrophages and DCs (Figure S9C, box 2) at the lesion adjacent normal, suggesting a less active immune microenvironment, as macrophages can suppress the antigen presentation of dendritic cells, subsequently impair CD8^+^ T‐cell responses [34].

To delineate the dynamics of comprehensive multicellular TME during the evolution of LUAD in two models, we derived 560 features, which were categorized into cell type (CT) composition (32), CT morphology (64), CT state (208), and CT interaction (256) of 17 major cell types (Figure 4B,C; and Tables S4 and S5). While the majority of features exhibited a parallel evolutionary pattern in both models, such as the increased B‐cell area and CD8 T‐cell area (morphology feature) from early to late stages (Figure 4D,E), some features such as DC density (composition feature) revealed distinct evolutions. Specifically, DC density increased and then decreased from normal to IAC in 129S4 K but consistently increased from normal to IAC in 129S4 U (Figure 4D,E). Given the important roles of DCs in antigen presentation, these findings may reflect the higher mutation burden (likely higher neoantigen burden) in the 129S4 U model.

### 2.6 TME Remodeling Throughout Tumor Development from Spatial Evolution Analysis

**FIGURE 5:**
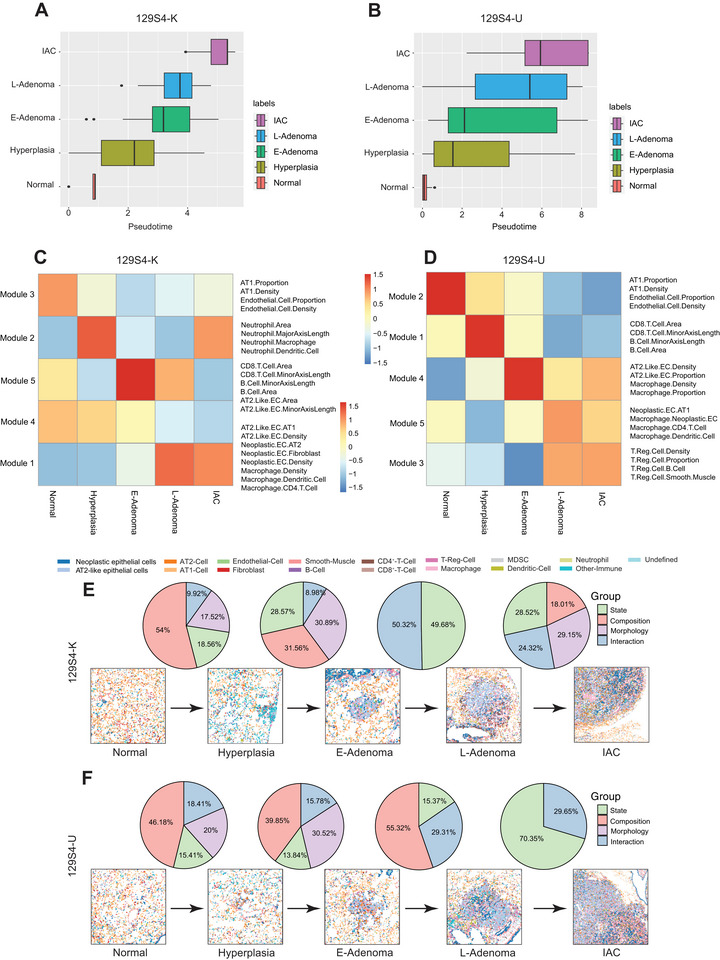
Coordinated changes in the TME mark the transition from normal to hyperplasia, E‐Adenoma, L‐Adenoma, and IAC. (A,B) Box plot view distribution of inferred pseudotime across five mice LUAD histological stages of (A) 129S4 K and (B) 129S4 U models. (C,D) Heat map of the distinguishing feature prevalence in normal, hyperplasia, E‐Adenoma, L‐Adenoma, and ADC samples. K‐means clustering separated features into five groups for (C) 129S4 K and (D) 129S4 U of distinct feature‐enrichment patterns in the tissues states, including those highest in each pathological lesion tissue and low in other stages. (E,F) Feature contribution analysis across five mice LUAD pathological stages of (E) 129S4 K and (F) 129S4 U models.

To comprehensively evaluate the molecular and immune features underlying early LUAD carcinogenesis, we depicted the evolution of the TME architecture as normal tissue progresses to IAC using Monocle 3 analysis [35], which revealed the primary trajectory across different histological stages in both models (Figure 5A,B). Next, we compared the composition, morphology, state, and interaction characteristics of the cellular tumor (CT) microenvironment across the normal, hyperplasia, E‐Adenoma, L‐Adenoma, and IAC stages, as previously defined (Figure 4B,C). Significant similarities were observed between the 129S4 U and 129S4 K models regarding the TME features associated with early carcinogenesis (Figure 5E,F). For example, during the transition from normal tissue to hyperplasia, composition features emerged as the most dominant in both models. At the subsequent transition from hyperplasia to E‐Adenoma, a combination of composition and morphology features became predominant. However, the TME evolution began to diverge between the two models at later stages. In the 129S4 K model, the transition from E‐Adenoma to L‐Adenoma was predominantly characterized by cell‐state‐related features and cell–cell interaction features. In contrast, morphology‐related features continued to play a major role in the 129S4 U model during this transition, although an increase in cell–cell interaction features was also noted. At the final transition from L‐Adenoma to adenocarcinoma, cell‐state‐related features dominated in the 129S4 U model. In the 129S4 K model, however, there appeared to be balanced contributions from composition, morphology, state, and interaction features. Overall, these findings suggest that cell state and interaction features—indicative of a more complex tumor microenvironment—may play increasingly prominent roles during the later stages of LUAD carcinogenesis.

### 2.7 Pathway Activities during the Tumor Evolution in Carcinogen‐Induced and Genetic LUAD Models

**FIGURE 6:**
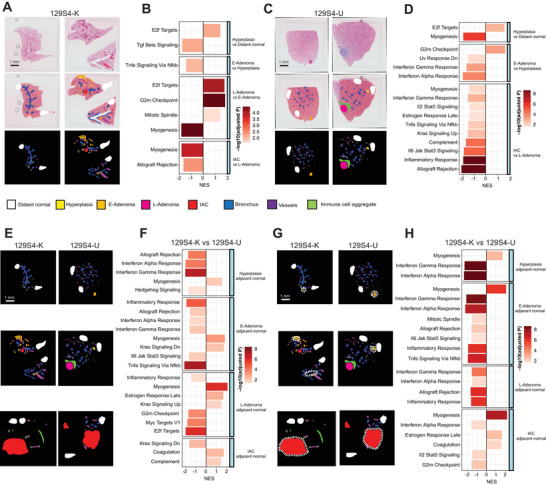
Spatial transcriptomics revealed the evolution transcripts difference between 129S4 K and 129S4 U models. (A) Pathological annotation of ST H&E images and spots mapping of the 129S4 K model, each color represents one type of ST spots area. Scale bars, 1 mm. (B) Hallmark pathway difference between stages from distant normal to hyperplasia to E‐Adenoma to L‐Adenoma to IAC of the 129S4 K model. (C) Pathological annotation of ST H&E images and spots mapping of the 129S4 U model; each color represents one type of ST spots area. Scale bars, 1 mm. (D) Hallmark pathway difference between stages from distant normal to hyperplasia to E‐Adenoma to L‐Adenoma to IAC of 129S4 U model. (E) Spots mapping of 129S4 K and 129S4 U models, yellow color denotes hyperplasia, orange color denotes E‐Adenoma, pink color denotes L‐Adenoma, and red color denotes IAC of two models. Scale bars, 1 mm. (F) Hallmark pathway difference between matched stages lesions inside of 129S4 K and 129S4 U at hyperplasia, E‐Adenoma, L‐Adenoma, and IAC stages. (G) Spots mapping of 129S4 K and 129S4 U models, white dotted line highlighted the adjacent normal area of each histological lesion. Scale bars, 1 mm. (H) Hallmark pathway difference between matched stages lesion adjacent normal of 129S4 K and 129S4 U at hyperplasia, E‐Adenoma, L‐Adenoma, and IAC stages.

Next, we systemically analyzed Visium spatial transcriptomics data from 156 LUAD and LUAD precursor samples that generated 51,531 transcript expression spots (Figure 6A–D and Table S6). Spot classification was modeled by aligning Visium images with pathologically annotated H&E images, resulting in eight defined spot types: normal, hyperplasia, E‐Adenoma, L‐Adenoma, adenocarcinoma, bronchus, vessels, and immune cell aggregate areas (Figure 6A–D).

In the 129S4 K model, pathway analysis (Figure 6A,B and Table S7) revealed downregulation of the Transforming Growth Factor‐beta (TGF‐β) pathway and upregulation of E2F target pathways in hyperplasia compared to normal lung tissue, indicating increased cell proliferation, DNA replication, and cell differentiation. The Tumor Necroses Factor‐alpha (TNF‐α) signaling pathway via Nuclear Factor kappa‐light‐chain‐enhance of activated B cells (NF‐κB) was uniquely upregulated in E‐Adenoma compared to hyperplasia (Figure 6B).

In the 129S4 U model (Figure 6C,D), similar to the 129S4 K model, hyperplasia showed an upregulation of E2F target pathways compared to normal lung tissue (Figure 6D). Notably, we observed a more active immune response in the early stages of the 129S4 U model compared to later stages, consistent with findings from IMC data (Figure 6D). Specifically, hyperplasia demonstrated elevated Interferon‐gamma (IFN‐γ) and Interferon‐alpha (IFN‐α) responses relative to E‐Adenoma, while L‐Adenoma showed higher IFN‐γ, inflammatory response, and Interleukin‐6 (IL‐6)/Jak/Stat3 signaling activities compared to IAC (Figure 6D). These results align with prior observations of greater immune infiltration during early stages of tumor development compared to later stages, as demonstrated by IMC data (Figure 3C and Figure S6C), indicating a progressive increase in immune suppression as the tumor advances.

Subsequently, we performed pathway analyses comparing the 129S4 K and 129S4 U models at matched histological stages (Figure 6E,F and Figure S10A). Overall, the 129S4 U model exhibited stronger IFN‐γ and IFN‐α responses at the hyperplasia and E‐Adenoma stages compared to the 129S4 K model (Figure 6F), consistent with the higher immune infiltration observed in 129S4 U by IMC profiling (Figure 3D). Similarly, IFN‐γ and IFN‐α pathway activities were elevated in the adjacent normal lung tissues at various stages in the 129S4 U model compared to the 129S4 K model (Figure 6G,H), corroborating the higher immune infiltration and active functional T‐cell states in these tissues (Figures S7C,I and S8A).

## 3 Discussion

Preclinical studies using animal models are essential in cancer research before testing therapeutic interventions in human trials. Given the substantial heterogeneity of human malignancies, it is crucial to test multiple models with diverse genetic backgrounds and/or etiologies. Many GEMMs and carcinogen‐induced models have been utilized in lung cancer research [17, 20, 36, 37, 38, 39, 40]. However, only a few animal models have been tested lung precancer evolution. Furthermore, the spatial TME architecture, an increasingly recognized critical feature for antitumor immunity, remains largely unknown in lung cancer precancer models. Previous studies examining mouse LUAD progression at the single‐cell level in the KrasG12D/+; P53R172/+ (KP) mouse model tumors revealed a lack of alignment between CNV patterns and transcriptional states of individual cells [9, 10]. This observation implies that the genetic drivers alone do not determine cell states during tumor progression; additional factors such as the TME and epigenetics also play significant roles [41].

In this study, we introduce a spatial atlas outlining the progression of Kras‐driven LUAD precancer GEMM 129S4 K and carcinogen‐driven LUAD precancer 129S4 U, resembling human LUAD precancer development and evolution in morphology, genomic features, as well as dynamic immune changes. Our objective was to explore the spatial locations of cells and their interactions within the TME as the tumor develops. Using WES, IMC, and ST on longitudinal mouse LUAD and LUAD precursor samples, we comprehensively characterized the complex TME landscapes along the evolutionary trajectory of these two mouse LUAD models. This approach allowed us to demonstrate the TME features and properties associated with the phenotypic progression of mouse LUAD. We identified unique cellular composition and spatial organization linked to mouse LUAD progression and outcomes, highlighting distinct molecular phenotypes and TME profiles between 129S4 K and 129S4 U.

In a recent spatial single‐cell study, the critical role of macrophages in the lung cancer environment was highlighted, but the analysis was confined to late stages of LUAD [42]. In our study, we observed a continuous increase in macrophage proportion and density during mouse Kras‐driven LUAD tumor progression (Figure 3), aligning with existing knowledge of macrophage involvement in lung cancer development and progression [43, 44, 45, 46]. Additionally, we noted an augmented interaction between macrophages and neoplastic epithelial cells as tumors progressed in two models (Figure 4).

Furthermore, our observations included increased proportions and density of immune‐suppressive Tregs, along with decreased proportions and density of antitumor immune CD8 T cells and B cells (Figure 3). Tregs inhibit the activity of other immune cells, such as cytotoxic T cells crucial for attacking and eliminating cancer cells [47]. Moreover, the cell–cell interaction between Tregs and neoplastic epithelial cells increased across different histological stages as tumor progression (Figure 4). These findings suggest progressive immunosuppression and remodeling of the tumor microenvironment favoring adaptations by tumor cells consistent with the findings from human LUAD precursors [14, 29].

RNA‐seq analysis of digested tissues struggles to distinguish between the five stages in mouse tissue, primarily due to limitations in lesion size. However, incorporating spatial information proves crucial in discerning distinct histological stages. By aligning Visium transcriptomics data with pathologically annotated H&E images, we unveiled the spatial pathway activities of two models across different histological stages and between the two models at matched lesion types (Figure 6). Hallmark pathway analysis results underscored that cell proliferation, DNA replication, and cell‐differentiation‐related pathways, such as E2F and G2M checkpoint, are the predominant distinguishing pathways across various stages. Notably, immune‐response activities were higher in the early histological stages especially in 129S4 U within matched lesion types, immune‐response activities both inside and outside the lesion were higher in 129S4 U than GEMMs. While Visium transcriptomics aids in distinguishing different histological types within small‐sized mouse lung tissue, its analysis remains confined to the bulk RNA‐seq level, necessitating further exploration at the subcellular transcriptomic level.

LUAD TMB and CNV of Kras‐driven 129S4 K and 129S4 U were compared in a recent study, though the analysis was confined to the latest histological stages [17]. In our investigation, we noted a continuous increase in both CNV and TMB in both mouse models, with TMB in 129S4 U significantly surpassing that in 129S4 K at matched histological types. Elevated TMB is frequently linked to heightened immune infiltration in the tumor microenvironment [48]. Tumors with increased TMB often harbor a greater number of neoantigens, unique proteins arising from these mutations [49]. TMB has emerged as a potential factor associated with the efficacy of immune checkpoint blockade (ICB) across various tumor types, including lung cancer [50, 51, 52]. Notably, high TMB in NSCLC is associated with increased immune infiltration and is relevant to ICB [30]. Syngeneic mouse models with higher TMB have demonstrated therapeutic benefits after ICB [53, 54]. Our results indicate that 129S4 U exhibits significantly higher immune cell density within the lesion across all histological stages compared to GEMMs, with particularly elevated density at the lesion interface, especially at the IAC stage (Figure S6). This is accompanied by increased cell–cell interactions between neoplastic epithelial cells and lymphoid cells in 129S4 U compared to 129S4 K (Figure 4). Furthermore, immune activities are higher in the lesion interior and at the lesion interface of 129S4 U compared to 129S4 K (Figure 6).

It is important to note that immune response and immune evasion are universal across different malignancies, including the two models discussed. However, the patterns of immune response and evasion can vary due to different etiologies and underlying molecular features. In the TMB/neoantigen‐high 129S4 U model, antigen/T‐cell‐centered immune response and evasion (e.g., higher antigen‐presenting cells such as DCs, higher immune effector CD8^+^ T cells, and higher T‐cell regulators such as Tregs compared to the 129S4 K model) may be more prominent. On the other hand, in the oncogene‐driven, TMB‐low, CNV‐high 129S4 K model, other non‐T‐cell‐centric antitumor immune responses and evasions (e.g., higher macrophage infiltration in the early stages compared to the 129S4 U model) may play more important roles. These findings suggest that the differences in the TME between 129S4 U and 129S4 K may have the potential to shape the immune response in lung cancer prevention and treatment. Both models are valuable for lung cancer interception preclinical studies, but they may be relevant for different targets. For instance, the 129S4 U model may be well suited for evaluating T‐cell–targeted interception strategies such as ICB given its higher levels of T‐cell engagement and immune checkpoint expression, while the 129S4 K model may be a good model to test immune interception agents targeting macrophages.

Animal models, including genetically GEMMs and carcinogen‐induced models, have played an essential role in cancer research and have become increasingly important in the era of immunotherapy, where an intact and competent TME is required [55]. Given their distinct molecular and immune characteristics, GEMMs and carcinogen‐induced models can serve complementary roles in studying cancer biology. However, these models also have important inherent limitations. First, most animal models do not fully recapitulate human tumors. In our study, although 129S4 U tumors exhibited molecular and immune features resembling human LUAD from smokers, their mutational spectra and signatures showed both shared and distinct features compared with smoking‐associated human LUAD, likely reflecting differences in genetic background and carcinogen exposure, as cigarette smoke contains thousands of chemical compounds [28]. Second, these models typically represent only specific molecular subtypes of human cancers. Lung cancers driven by different oncogenic alterations can display markedly distinct molecular and immune features [56, 57, 58] and even within KRAS‐mutant LUAD, different KRAS variants (e.g., G12D, G12C, G12A, G13R, and Q61H) and co‐mutation patterns can confer divergent biological and clinical phenotypes [59, 60]. Accordingly, our findings should be viewed as a proof of principle for model selection and hypothesis generation. Third, due to homogeneous genetic backgrounds, shorter life spans, and accelerated carcinogenesis in mice, these models often lack the intra‐ and intertumoral heterogeneity and molecular complexity characteristic of human tumors. While these features offer practical advantages—such as rapid phenotypic readouts and smaller sample size requirements—results must be interpreted with caution and may not be broadly generalizable. Therefore, integrating animal models with complementary human‐relevant systems, including cancer cell lines, organoids, and patient‐derived xenografts, will be essential for studying human cancer biology more comprehensively.

In summary, our results offer valuable insights into alterations in spatial cellular composition, organization, and molecular profiles across different histological lesions representing mouse LUAD. The development of an early‐spectrum mouse LUAD atlas will significantly enhance our understanding of disease pathogenesis. As lung cancer prevention and precancer interception clinical trials emerge in oncologic research (NCT04789681 [61]; NCT03634241; and NCT03347838), these findings will be of great value to guide preclinical studies testing novel interception strategies.

## 4 Methods

### 4.1 Mouse Models

129S4 Kras^G12D^ (K) (No: 008180) and 129S4 wild‐type mice (No: 009104) were purchased from Jackson Laboratory. All animal housed in colony cages under pathogen‐free conditions at MD Anderson Research Animal Support Facility. The mice were kept at an ambient temperature of 20°C –26°C and a humidity range of 30%–70% with a 12‐h light–dark cycle. All animal experiments were conducted following Institutional Animal Care and Use Committee‐approved protocols (Approval number: 00001217‐RN03). For carcinogen‐induced mouse models (CITMs), we used a urethane‐induced mouse model. Specifically, 129S4 wild‐type mice received intraperitoneal injections of urethane at a dosage of 1 mg/g (body weight) three times over 8 days when they were 6 weeks old. 81 mice were sacrificed at 1, 2, 4, 7, 14, 20, 30, and 40 weeks after urethane administration, with a 0‐week timepoint for mice that received no treatment. We collected both normal lung and lung tumor tissues for downstream analysis. For 129S4 K GEMM, 60 mice were intranasally instilled with 2.5 × 10^7^ pfu of Ad5‐CMV‐Cre recombinase adenovirus at the age of 6‐week‐old, and sacrificed at 1, 2, 4, 7, 9, 15, 20, and 30 weeks after adenovirus administration, with a 0‐week timepoint for mice that received no adenovirus administration. We collected both normal lung and lung tumor tissues for downstream analysis.

### 4.2 Antibody Panel Creation and First Step Validation IMC Antibody Conjugation

An antibody panel was designed to specifically target epitopes associated with mouse lung cancer, in addition to markers for cell cycle regulation and immune checkpoints. This panel (Table S1) was also designed to distinguish between epithelial, stromal, and immune cell types. Clone information is available in Table S1. To ensure the reliability of these antibodies, a series of validation steps were carried out. Initially, all antibodies listed in Table S1 underwent testing via immunohistochemistry (IHC) (Figure S2). For immune markers, this testing involved a tissue microarray (TMA) constructed using various lymphoid tissues, including spleen, thymus, and lymph nodes. Lung cancer tissues were used (Figure S3A) for markers related. Subsequently, a qualified pathologist reviewed the results of these tests to ensure that the expression patterns matched existing literature and that the signal intensity was consistently high. Only antibodies meeting these criteria successfully passed our quality control assessment.

### 4.3 IMC Antibody Conjugation and Second Step Validation

Antibodies passed the initial validation were subsequently labeled with metals using the MaxPar X8 Multimetal Labeling Kit (Standard BioTools) following the manufacturer's recommended protocol. After the conjugation process, all antibodies underwent reevaluation by IMC to ensure that their specificity remained intact despite the labeling. The IMC staining results were subjected to validation by another experienced pathologist. To determine the optimal antibody concentrations, we conducted tests using various concentrations for each antibody. Finally, we applied the complete antibody panel to stain tissue arrays containing lymphoid tissues and various types of cancer tissues, validating both positive and negative staining patterns.

### 4.4 Preparation and Staining

Formalin‐fixed paraffin‐embedded (FFPE) slides were deparaffinized at 56°C overnight in an oven (Thermo Scientific). Tissue section rehydration was carried out in a fume hood with the following steps: three washes with metal‐free xylene, followed by two washes with 100% metal‐free ethanol, two washes with 95% metal‐free ethanol, two washes with 70% metal‐free ethanol, one wash with metal‐free ddH_2_O, and one wash with metal‐free phosphate‐buffered saline (PBS), each for 10 min. The antigen or epitope retrieval is performed with EZ‐Retriever v 3.0 microwave. A solution containing 250 mL of 1× volume for the holder with Dako target retrieval pH9 (10×; Ref.S2367) was prepared. The microwave was set to reach 99°C for 10 min and repeated for two cycles. The slide tank was removed from the microwave and left at room temperature for 15 min after antigen or epitope retrieval. Subsequently, slides were washed with metal‐free ddH_2_O twice and metal‐free PBS once. Slides were stained with a cocktail containing metal‐tagged antibodies and maintained in a humidified chamber overnight at 4°C. All conjugations were performed by the Department of Translational Molecular Pathology at MD Anderson Cancer Center (MDACC) using Maxpar Conjugation Kits (Standard BioTools). Information about the antibodies used is available in Table S1. Following staining, slides were washed with metal‐free PBS three times, each for 5 min. Nuclei were counterstained with a 0.625 µm isidium solution at room temperature for 30 min, followed by three washes with metal‐free PBS each for 5 min. Tissue structures were counterstained with 0.0005% ruthenium at room temperature for 4 min and then washed with metal‐free PBS three times each for 5 min. Dehydration of the slides was achieved with two washes of metal‐free ddH_2_O, followed by one wash each of metal‐free 70% ethanol and metal‐free 100% ethanol. The slides were then air‐dried under a hood for at least 15 min and stored properly at 4°C before and after data acquisition. Tissue ablation was performed using the Standard BioTools Hyperion imaging mass cytometer in the MDACC North Campus Flow Cytometry and Cellular Imaging Core Facility.

### 4.5 ROI Selection

ROIs were selected on H&E‐stained sections by an experienced pathologist using clear histomorphologic criteria, focusing on viable tumor and representative stromal or interface areas while avoiding artifacts, tissue edges, and overt necrosis or fibrosis. ROI selection was performed blinded to experimental groups. Balance checks across batches, sections, and operators were conducted by comparing ROI characteristics, and any detected imbalances were accounted for in downstream analyses.

### 4.6 IMC Imaging Acquisition

Data acquisition was carried out using a Helios time‐of‐flight mass cytometer (CyTOF) in conjunction with a Hyperion Imaging System (Standard BioTools). Prior to laser ablation, optical images of the slides were obtained using the Hyperion software, and the specific areas for ablation were chosen as previously described. Laser ablation was performed with a resolution of approximately 1 µm and a frequency of 200 Hz. To ensure consistent performance, the machine underwent daily calibration using a tuning slide that included five metal elements (Standard BioTools). In total, we acquired 290 ROIs. Four ROIs were excluded due to data corruption, one due to inadequate image quality, and one because the areas lacked islets. All subsequent analyses were conducted using the remaining 284 ROIs.

### 4.7 IMC Data Preprocessing

The acquired IMC data were first converted to TIFF format using the MCD viewer (Standard BioTools) for further analysis. To mitigate channel crosstalk occurring in mass cytometry experiments due to minor isotopic impurities, we initially immobilized all metal‐conjugated antibodies onto an agarose‐coated glass slide. Subsequently, we quantified the isotopic composition through IMC. To address the issue of crosstalk, we harnessed the Bioconductor CATALYST [62] package to generate a “spillover matrix” from this dataset (Table S2). This matrix enabled us to apply a non‐negative least‐squares regression model via CATALYST [62] for cross‐channel spillover correction in single‐cell expression data. Furthermore, we performed noise reduction by filtering out sparse and pixelated signals. Additionally, we conducted aggregate filtering to remove antibody aggregates recognized as minuscule, connected components in the image.

### 4.8 Single‐Cell Segmentation

Cell segmentation was performed on preprocessed images using deep learning‐based software Mesmer published by Greenwald et al. [63]. The input to Mesmer is a two‐channel image containing a nuclear marker and a membrane or cytoplasmic marker to accurately delineate single cell boundaries. We adopted Ir191 and double‐stranded deoxyribonucleic acid (dsDNA) as the cell nuclear channel and a combination channel of Pan‐CK, B2M, and NakATPase as the membrane channel, as input for Mesmer. To more effectively capture the range of cell shapes and morphologies present in LUAD, we generated two distinct segmentation parameter sets optimized for nonepithelial and epithelial cells, and further combined the outcome for the final cell segmentation. The nonepithelial setting used a radial expansion of two pixels from the detected nuclear border to generate cell objects, and a stringent threshold for splitting cells. The epithelial setting used a radial expansion of three pixels and a more lenient threshold for splitting cells. We then combined these masks using a postprocessing step that gave preference to the epithelial segmentation objects, overriding stromal‐parameter‐detected objects in the same area.

### 4.9 Cell Clustering

Cell clustering was performed in two stages: building an integrated reference dataset using subset of the original data and annotating the rest of the data using this reference. First, we identify two batches of data and integrate them into a shared reference so that cells from the same group will cluster together. Specifically, we used all cell markers to find anchors between the two batches by determining a shared low‐dimensional space using canonical correlation analysis (CCA). The identified anchors are used to construct the integrated dataset according to the strategy outlined in [35]. We next standardized expression values for the cell markers and select only the lineage cell markers including CD326, Pan‐CK, TTF‐1, SPC, RAGE, Vimentin, a‐SMA, CD31, CD45, CD3e, CD4, FoxP3, CD8a, PAX5, CD11b, CCR2, ARG, Ly6G, CD11c, and F4/80 to prioritize for cell clustering. The Louvain community detection algorithm was then applied on principal components determined on the selected cell markers resulting in 17 clusters. To assign descriptive labels to these clusters, we used mean marker expression and determine cell types belonging to two general groups (tumor cells and immune cells). Here we used 17 function markers including CD44, ECAD, ICOS, PD‐L1, CTLA4, TIM‐3, Granzyme‐B, CD25, iNOS, CD127, Ki‐67, CD21, and CD206. After determining the cell types for the references data, we map the individual query sets onto the reference and transfer the cell‐type labels from the reference to the query sets using the method described in [35].

### 4.10 Cell–Cell Pairwise Interaction

Because the distance between the nucleus of neighboring cells varies depending on their size and the degree of cellular agglomeration, we adopted the Delaunay triangulation [64, 65] to identify pairs of cells that are most likely in physical contact. We modeled the cell interaction network based on graph theory [62], with vertices representing cells and edges representing direct contacts between cells. Because the contact frequencies between different cell types are correlated with these cell‐type proportions, we used two parallel methods to obtain contact enrichment scores. A value of 1 would indicate perfect assortative mixing, where contacts occur only between cells from the same type. A value of 0 would indicate random mixing between cell types. A negative value would indicate disassortative mixing, a preference for contacts between cells from different types.

### 4.11 Neighborhood Identification

To generate cellular neighborhoods (CN), we used the “window capturing” strategy, which consists of the number of cells (*n*) in closest proximity to a given cell as described [14]. Each window then was represented as a frequency vector consisting of the types of X (as indicated) closest cells to a given cell. After obtaining all the window vector for each cell, cells were clustered using Scikit‐learn (a software machine‐learning library for Python) Minibatch K Means clustering algorithm (version 0.24.2) with default batch size = 100 and random state = 0. Every cell was subsequently assigned to a CN type based on its window vector. The prevalence of each neighborhood in each core was normalized so that the sum of neighborhood prevalence for that core was 1.0. Values were then *z*‐scored and cores with a *z*‐score above or equal to 0 and below 0 were compared for survival outcomes.

### 4.12 Feature Extraction

Based on segmented and recognized cells inside each ROI, we extracted four groups of features (Table S3): CT/CN composition (proportion and density), CT/CN morphology, CT/CN state, and CT/CN interaction. The CT/CN proportion of each ROI was measured by dividing the number of one cell subtype by the total number of cells inside, which assessed the cells’ relative abundance. The CT/CN density of each ROI was measured by dividing the number of one cell subtype by the occupied area, which evaluated the compactness of cells. Both CT/CN proportion and density (composition) were independent measurements of one cell subtype without consideration of the spatial arrangement among different cell subtypes which described in our previous study [66]. Morphological features, including area, eccentricity, major and minor axis lengths, were measured for investigating CT/CN morphologies, measured by the steinbock Python package (version v0.16.0). The CT/CN state features was evaluated by each CT/CN functional markers’ average expression per cell. CT/CN interaction quantifications were performed as previous elaborated in the cell–cell pairwise interaction, and interaction feature were extracted based on their interaction values at ROIs’ level.

### 4.13 Trajectory Analysis

For trajectory analysis, we used Monocle 3 [67]. First, samples were processed using the standard Seurat approach which include scaling, normalization, variable feature selection and Principal Component Analysis (PCA) analysis. We used Monocles default parameter settings to ensure that our trajectory analysis results are reproducible. An embedding of the samples into a Uniform Manfold Approximation and Projection (UMAP) subspace is then determined from which the trajectory graph was inferred. To model the evolution of tumor from one stage to another, we used domain knowledge and set the starting of the trajectory to be the cluster containing the normal samples. Finally, to further determine the features that govern the evolution of tumor from one stage to another along the trajectory, we test features for differential expression and find modules of co‐expressed genes across the trajectory.

### 4.14 Feature Importance Analysis

To identify differentially expressed (DE) features that facilitate tumor evolution from normal to AAH, AAH to AIS, AIS to MIA and MIA to IAC, we perform DE analysis by comparing this pairs of groups using the Wilcoxon rank‐sum test [68]. We set a cutoff such that DE analysis was performed only on features that associates with at least 25% of samples in both the compared stages. Feature importance were then computed using the returned adjusted *p*‐values of all DE features as

$$f_{i}=-\frac{\log\left({\bar{p}}_{i}\right)}{\sum_{k=1}^{n}\log\left({\bar{p}}_{k}\right)}$$

where ${\bar{p}}_{i}$ denotes the adjusted *p*‐value for feature *i* returned from the DE test.

### 4.15 Tissue Preparation and Spatial Transcriptomics

Normal and tumor tissues from mouse lungs were fixed in 10% formalin at room temperature for 24–48 h (using a fixative volume 5–10 times that of tissue volume). Fixed tissues were transferred to 70% ethanol for temporary storage at 4°C. Paraffin embedding was conducted by the MDACC Research Histology Core Laboratory. FFPE blocks were cut into 5 µm thick sections using a precooled, RNase‐free microtome. These sections were then transferred onto Visium Spatial Gene Expression slides (10× Genomics), which were pretreated by floating them on a water bath at 43°C. Following sectioning, the slides were dried at 42°C in a Thermal Cycler (SimpliAmp Thermal Cycler, Thermo Fisher Scientific) for 3 h, following the manufacturer's instructions. The slides were placed in a slide mailer, sealed with paraffin, and stored overnight in a refrigerator at 4°C. The slides were then deparaffinized, fixed, stained with H&E, and imaged at 5× magnification using a Leica DM5500 B microscope (Leica Microsystems). Tile scans of the entire array were acquired using the Leica Application Suite X (LAS X) and merged. Spatial gene expression libraries were processed according to the manufacturer's instructions (10× Genomics, Visium Spatial Transcriptomic) and sequenced using a NovaSeq 6000 sequencer (Illumina). All H&E staining, imaging, library preparation, and sequencing processes were carried out by the Genomic & RNA Profiling Core at Baylor College of Medicine.

### 4.16 Statistical Analysis

Statistical analyses were performed using GraphPad Prism (v.10) (for experimental data), and R (v.4.2.2), RStudio (v.2023.09.1) and Python (v.3.10.2) (for IMC data, ST data and matched clinical variables). Student's *t*‐tests, Wilcoxon rank‐sum tests and Analysis of Variance (ANOVA) were used for continuous variables. Paired *t*‐tests were used for paired comparisons. *p* < 0.05 was statistically significant.

## Author Contributions

J.J.Z. and B.Z. conceived the study. J.J.Z. and B.Z. wrote the manuscript. J.J.Z. and J.W. jointly supervised the study. J.F.J., B.Z., and F.R.R. supervised pathological assessments and the preparation of specimens. B.Z., O.S., L.M.S.S., and P.C.E.R. validated the IMC antibodies and stained specimens. P.J.C., J.R.L., and M.A. performed all bioinformatics and statistical data analyses. B.Z., P.J.C., M.A., J.W., and J.J.Z. interpreted the data. All authors reviewed and approved the manuscript.

## Conflicts of Interest

The authors declare no conflicts of interest.

## Declaration of Interests

J.J.Z. reports research funding from Merck, Johnson and Johnson, Novartis, Summit, Hengenix and consultant fees from BMS, Johnson and Johnson, AstraZeneca, Geneplus, OrigMed, Innovent, Varian, Catalyst outside the submitted work. I.I.W. reports Honoraria from Genentech/Roche, Bayer, Bristol‐Myers Squibb, Astra Zeneca/Medimmune, Pfizer, HTG Molecular, Asuragen, Merck, GlaxoSmithKline, Guardant Health, Oncocyte, Flame, and MSD; Research support from Genentech, Oncoplex, HTG Molecular, DepArray, Merck, Bristol‐Myers Squibb, Medimmune, Adaptive, Adapt immune, EMD Serono, Pfizer, Takeda, Amgen, Karus, Johnson & Johnson, Bayer, Iovance, 4D, Novartis, and Akoya. J.V.H. reports honorariums from AstraZeneca, Boehringer‐Ingelheim, Catalyst, Genentech, GlaxoSmithKline, Guardant Health, Foundation medicine, Hengrui Therapeutics, Eli Lilly, Novartis, Spectrum, EMD Serono, Sanofi, Takeda, Mirati Therapeutics, BMS, BrightPath Biotherapeutics, Janssen Global Services, Nexus Health Systems, EMD Serono, Pneuma Respiratory, Kairos Venture Investments, Roche and Leads Biolabs. The other authors declare no competing interests.

## Lead Contact

Further information and requests for resources should be directed to and will be fulfilled by the lead contact Jianjun Zhang (jzhang20@mdanderson.org).

## Supporting information




**Supporting File 1**: advs73897‐sup‐0001‐SuppMat.pdf.

**Supporting File 2**: advs73897‐sup‐0002‐TableS1‐S7.zip.

## Data Availability

All the original data presented in main are publicly accessible at Synapse (https://www.synapse.org/Synapse:syn64500279/files/). All software programs used for analyses are publicly available and listed in the key resources table.
