# Supplementary material for: Spatial Profiling Reveals Distinct Molecular and Immune Evolution of Mouse Lung Adenocarcinoma Precancers with or Without Carcinogen Exposure
Source: Adv Sci (Weinh). 2026 Jan 25;13(17):e12597. doi: 10.1002/advs.202512597 (PMC13042775; doi:10.1002/advs.202512597)
Supplement: Supplementary file 1 — Supporting File 1: advs73897‐sup‐0001‐SuppMat.pdf. [file ADVS-13-e12597-s002.pdf]

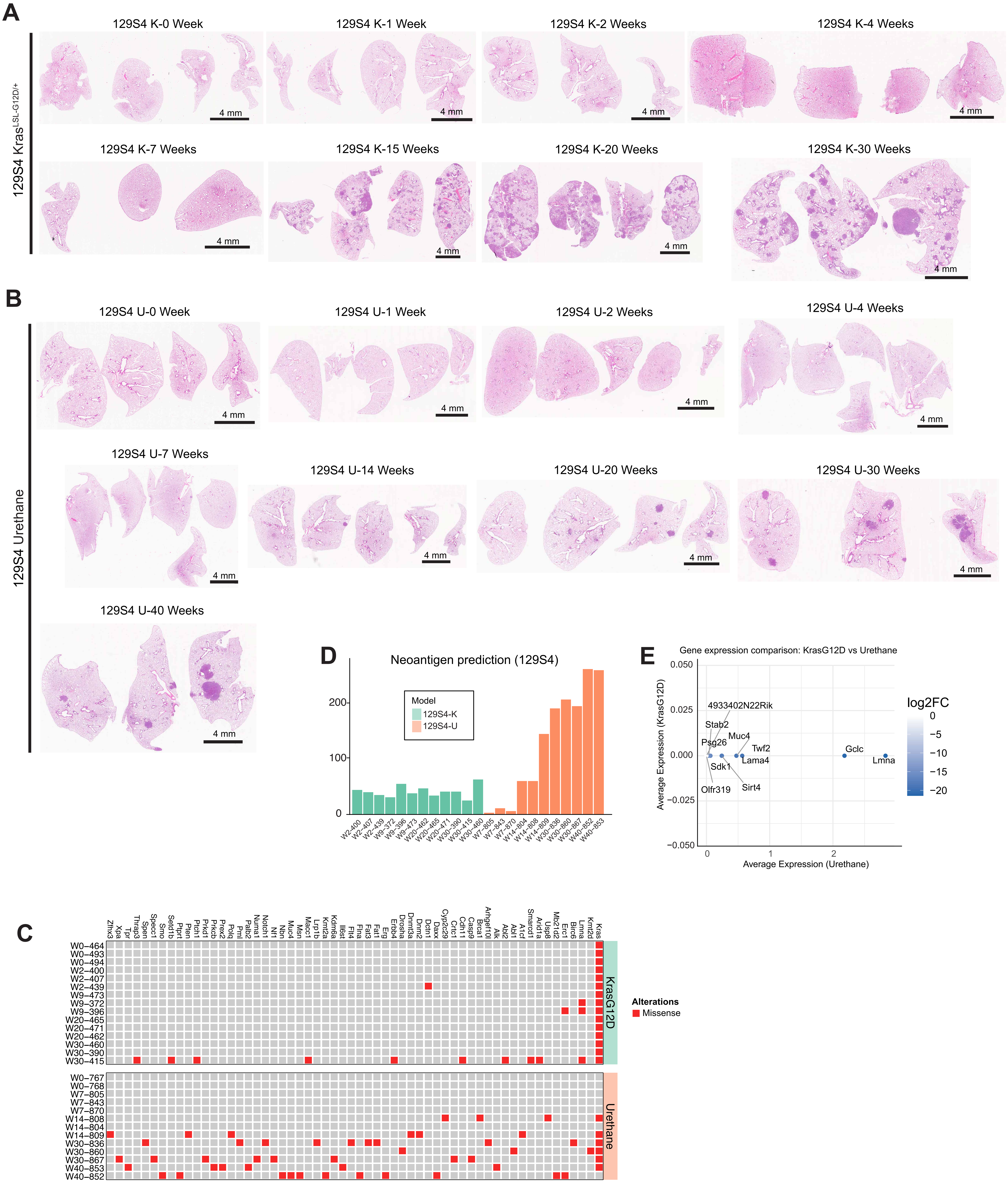

Extended Data Fig. 1 | Two precancer mouse models establishment and WES profiling.

A) Induction of LUAD precancerous and cancerous lesions in mice. 129S4 K model mice were induced with $5x10^7$ Ad5CMVCre Virus/mouse. Mice were sacrificed before (week 0), and after virus induction (weeks 1, 2, 4, 7, 9, 15, 20, 30), respectively, H&E staining identified 5 pathological lesions, Normal, Hyperplasia, E-Adenoma, L-Adenoma, and Adenocarcinoma.

B) 129S4 U model mice were induced with Urethane 1mg/g (body weight) 3 times/mouse in 1 week. Mice sacrificed before(week 0), and after Urethane induction (weeks 1, 2, 4, 7, 14, 20, 30, 40), similar pathological lesions were identified by H&E staining. Scale bars, 300um.

C) All missense (Mis), nonsense (Non) SNVs, synonymous in genes listed are displayed.

D) Neoantigen burden of 129S4 U and 129S4 K models.

E) The overlap neoantigen genes expression comparison between 129S4 U and 129S4 K models.

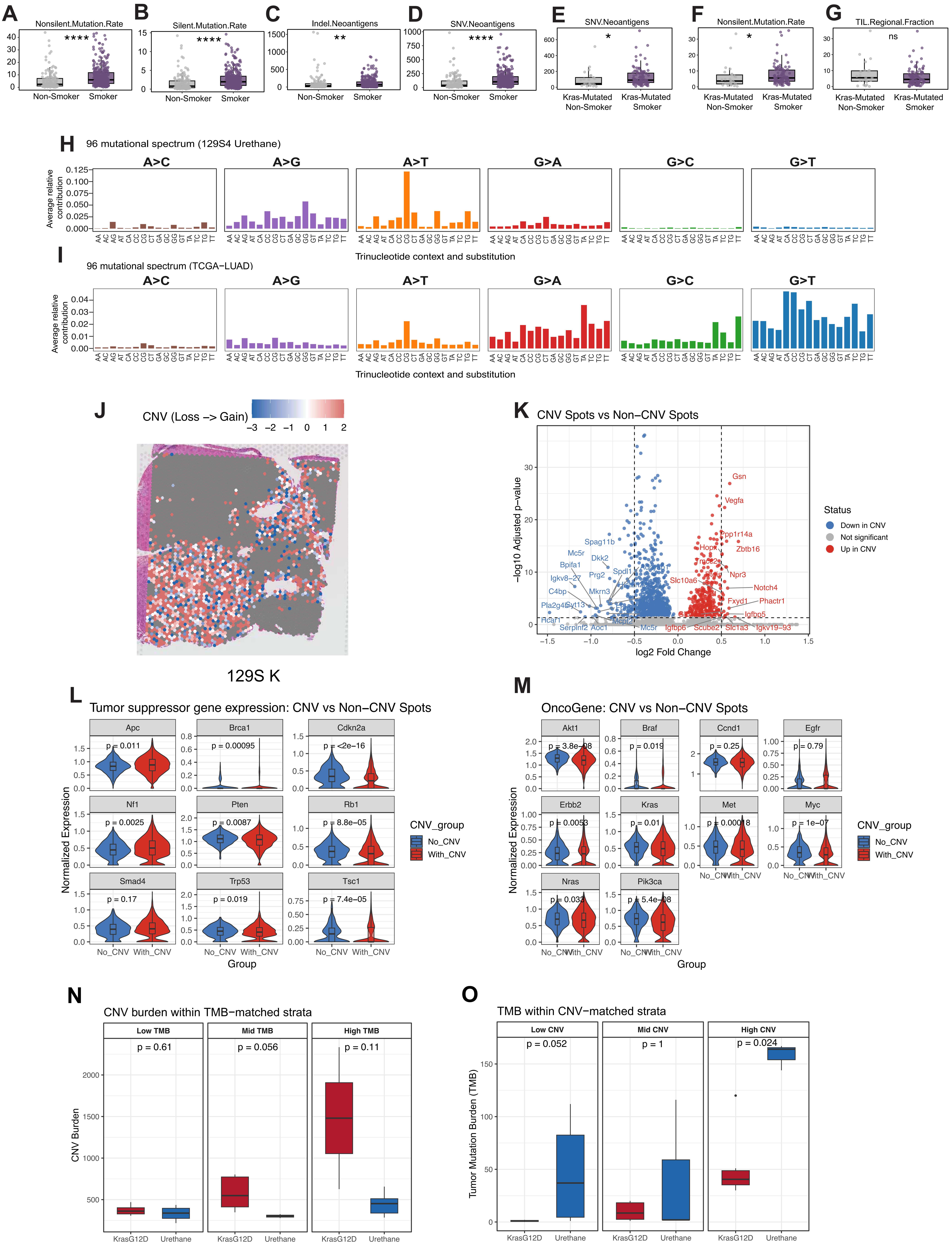

Extended Data Fig. 2 | Highly specific mutation signatures and CNV related genes distribution of 129S4 K and 129S4 U models.
A,B,C,D) Nonsilent mutation rate (A), silent mutation rate (B), indel neoantigens (C), and SNV neoantigens (D) between smoker and non-smoker of TCGA data.
E,F,G) SNV neoantigens (E), Nonsilent mutation rate (F), TIL Regional Fraction (G) between smoker and non-smoker of Kras-mutated smoker and non-smoker of TCGA data.
H) All 96 substitutions in 129S4 Urethane-induced mouse model.
I) All 96 substitutions in TCGA-LUAD data.
J) Representative spatial CNV visualities on tissue from 129SK model.
K) Gene enrichment of CNV high vs CNV low regions.
L) Tumor suppressor gene expression of CNV high vs CNV low regions.
M) Tumor oncogene expression of CNV high vs CNV low regions.
N) CNV burden between Urethane and KrasG12D tumors within TMB-matched strata.
O) TMB levels between models within CNV-matched strata.

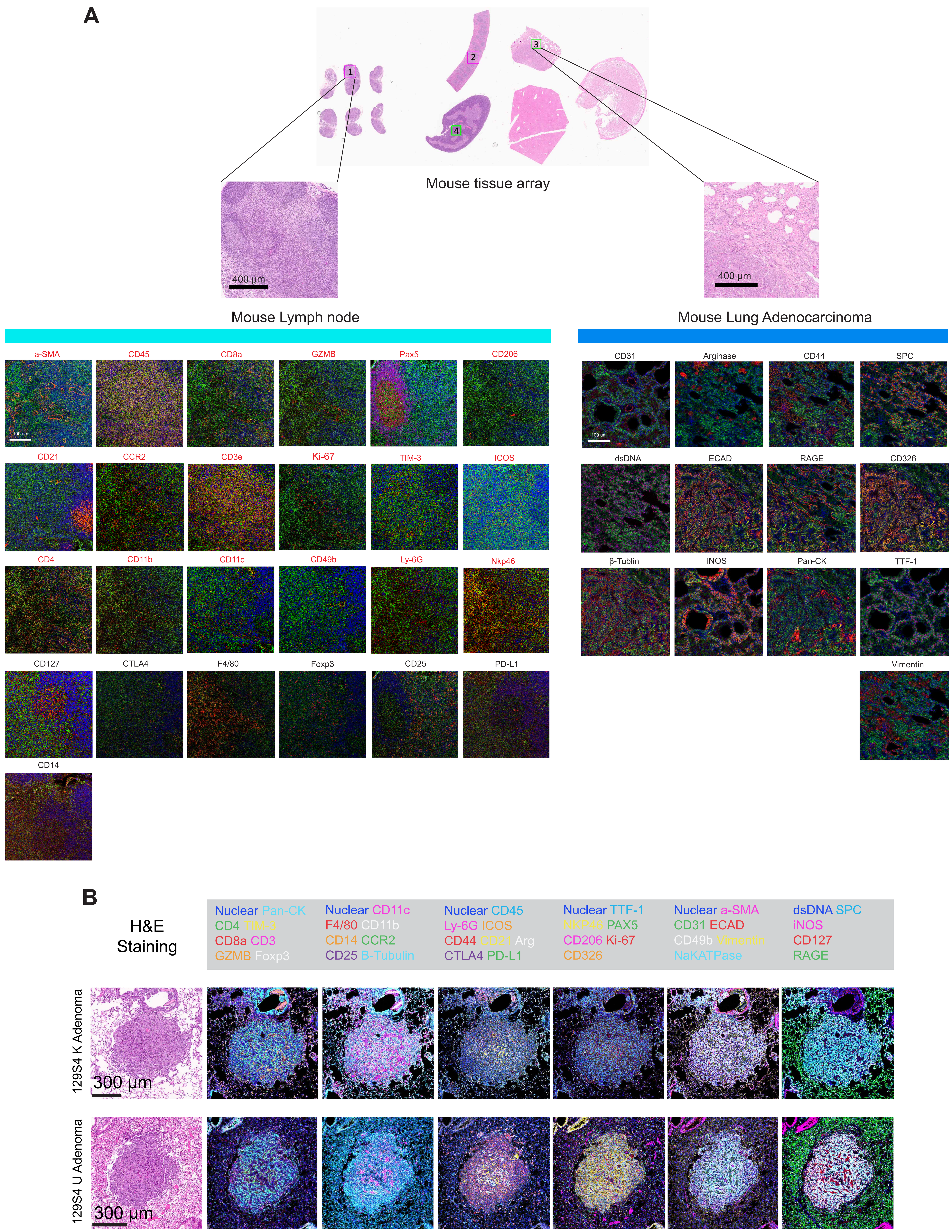

Extended Data Fig. 3 | Imaging mass cytometry antibody validation
A) Validation of 39 antibodies used for multiplex IMC across positive and negative controls. Nuclear (blue), Na-K-ATPase (green), marker in each channel (red), Scale bars, 100 µm or 400 µm.
B) Representative region of interest (ROIs) IMC staining images of 129S4 K and 129S4 U models, each color represents one marker staining. Scale bars, 300 µm.

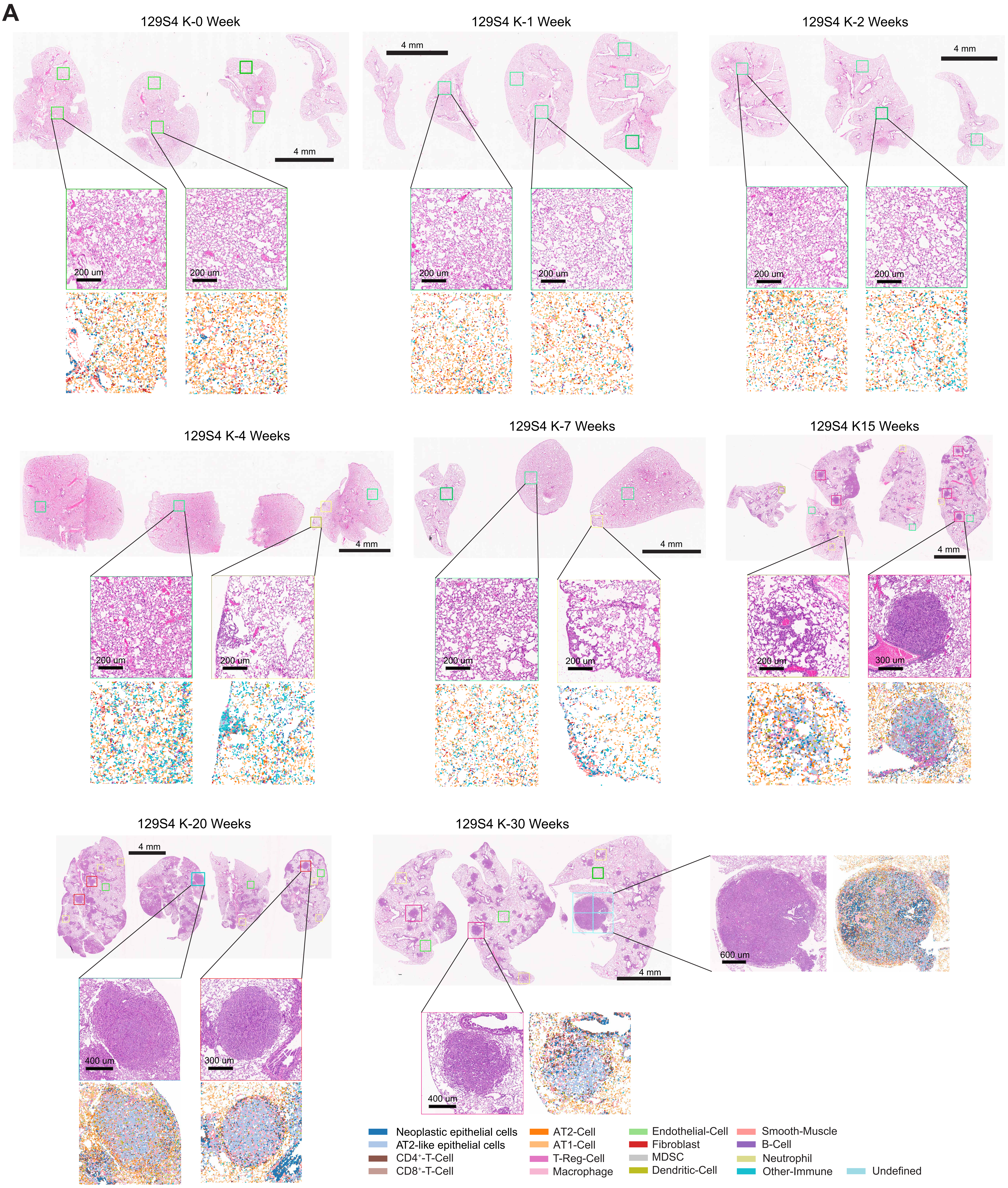

Extended Data Fig. 4 | Pathological ROIs selection strategy and different cell types of spatial location of 129S4 K model.
A) Representative cases from each sacrifice timepoints of 129S4 model showing pathological ROIs selection strategy, green and yellow square represents 800 x 800 μm scale ROI, red square represents 1 200 x 1200 μm scale ROI, and blue square represents 1500 x 1500 μm scale ROI. Pseudo color images donate the spatial localization of 17 major cell types, each color refer to the bottom represents one cell type. Scale bars, 200μm, 300μm, 400μm, 600μm, and 4mm.

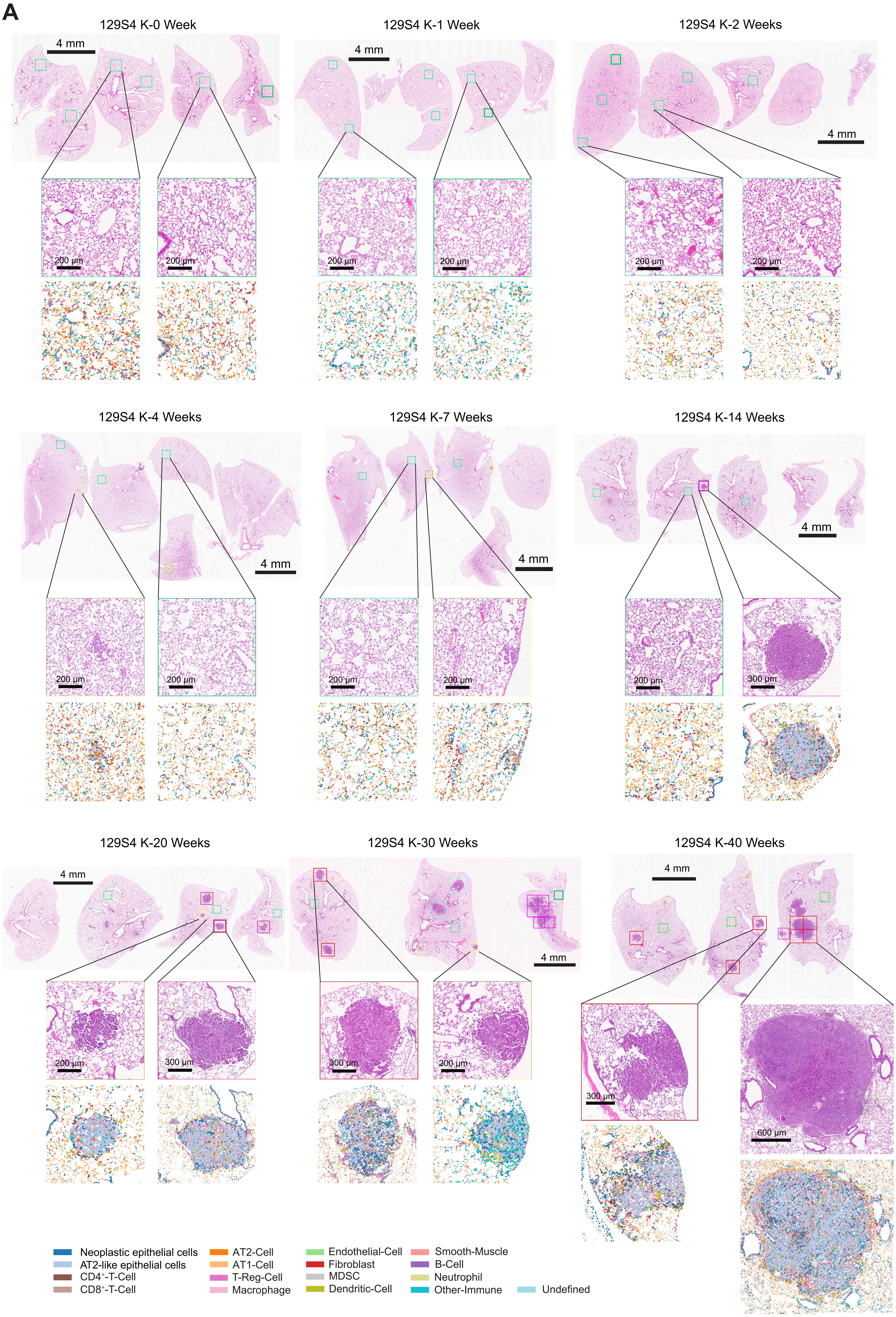

Extended Data Fig. 5 | Pathological ROIs selection strategy and different cell types of spatial location of 129S4 U model
A) Representative cases from each sacrifice timepoints of 129S4 U model showing pathological ROIs selection strategy, green and yellow square represents 800 x 800 μm scale ROI, red square represents 1200 x 1200 μm scale ROI, and blue square represents 1500 x 1500 μm scale ROI. Pseudo color images donate the spatial localization of 17 major cell types, each color refer to the bottom represents one cell type.
Scale bars, 200μm, 300μm, 600μm and 4mm.

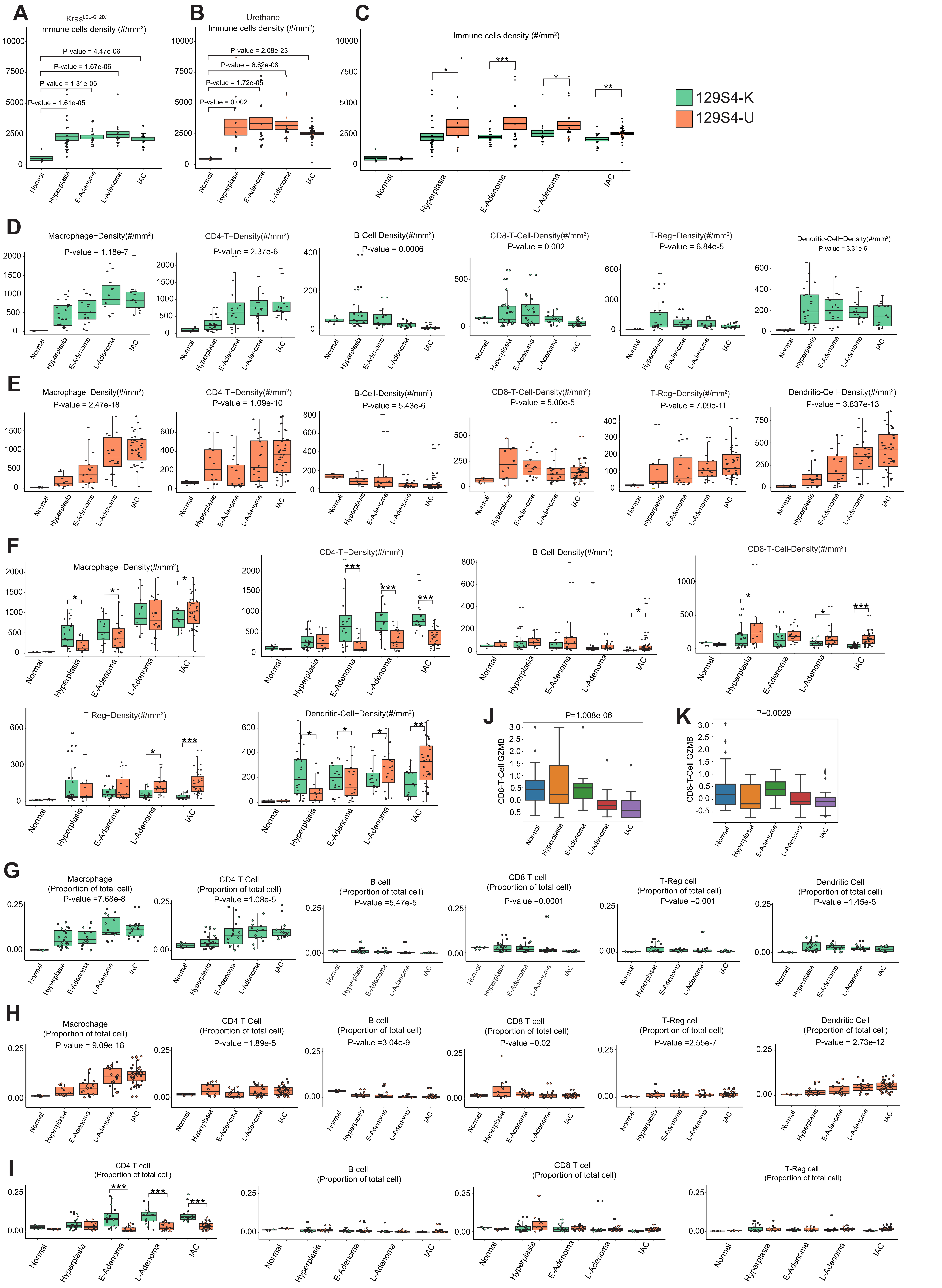

Extended Data Fig. 6 | Lesion inside immune cell proportion and density evolution, and comparison of 129S4 K (green color) and 129S4 U (orange color) models

A, B) Lesion inside overall immune cell density across five histological stages of 129S4 K (green color) and 129S4 U (orange color) models. (Pairwise multiple Welch's t-tests with Bonferroni correction).
C) Lesion inside overall immune cell density comparison between 129S4 K (green color) and 129S4 U (orange color) models at matched stages. (* p<0.05, ** p<0.01, and *** p<0.001, Two-tailed student's test).
D,E) Lesion inside immune cell subtypes density across five histological stages of 129S4 K (green color) and 129S4 U (orange color) models. (Pairwise multiple Welch's t-tests with Bonferroni correction).
F) Lesion inside immune cell subtypes density comparison between 129S4 K (green color) and 129S4 U (orange color) models at matched stages. ((* p<0.05, ** p<0.01, and *** p<0.001, Two-tailed student's test).
G,H) Lesion inside immune cell subtypes proportion of total cell across five histological stages of 129S4 K (green color) and 129S4 U (orange color) models. (Pairwise multiple Welch's t-tests with Bonferroni correction).
I) Lesion inside immune cell subtypes propotion of total cell comparison between 129S4 K (green color) and 129S4 U (orange color) models at matched stages. (* p<0.05, ** p<0.01, and *** p<0.001, Two-tailed student's test).
J) CD8-T-Cell GZMB expression value across pathological lesions of 129S4 K (J) and 129S4 U (K) models. (Pairwise multiple Welch's t-tests with Bonferroni correction).

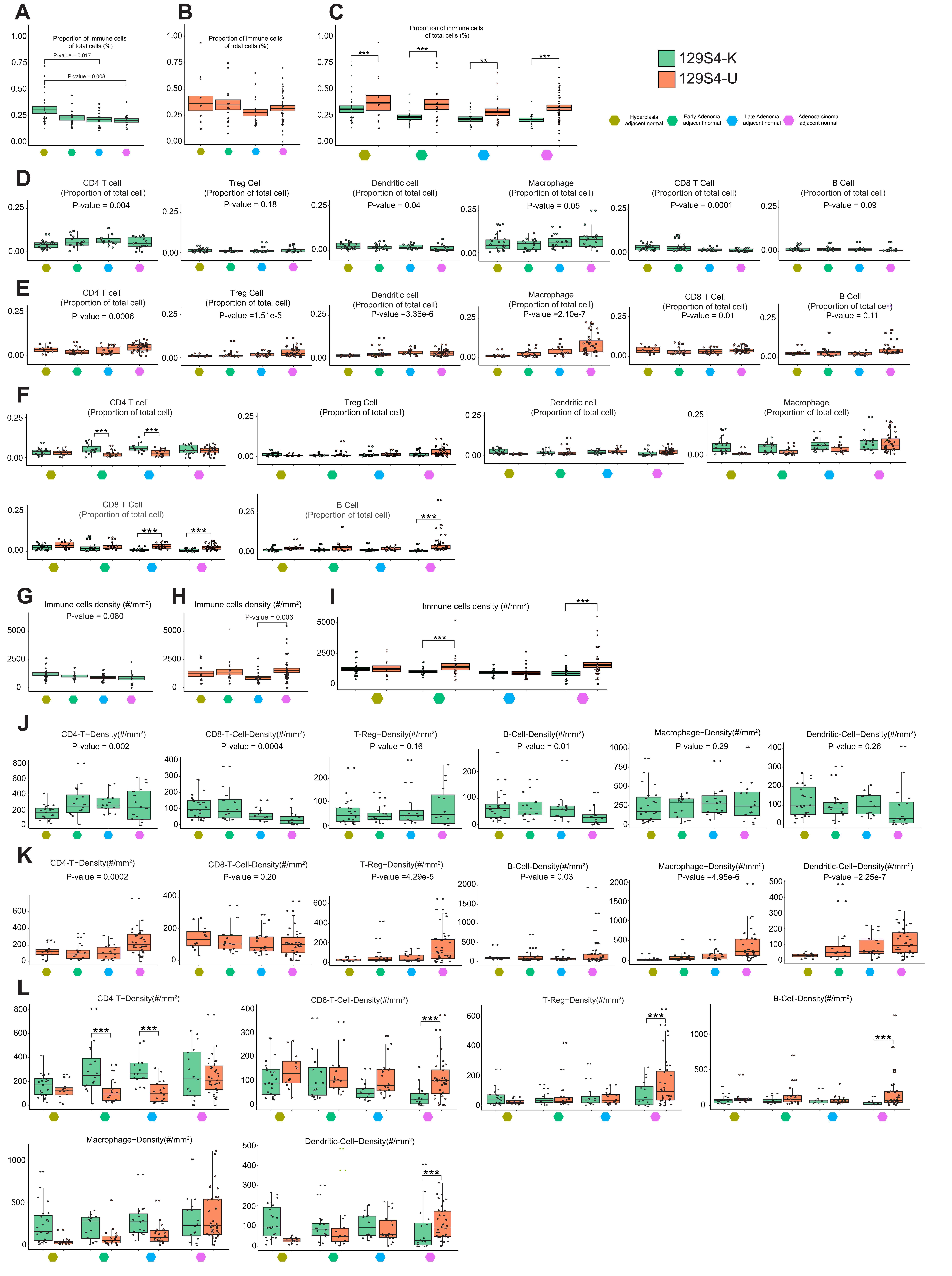

Extended Data Fig. 7 | Lesion adjacent normal immune cell proportion and density evolution, and comparison of 129S4 K (green color) and 129S4 U (orange color) models

A, B) Lesion adjacent normal overall immune cell proportion of total cell across five histological stages of 129S4 K (green color) and 129S4 U (orange color) models. (Pairwise multiple Welch's t-tests with Bonferroni correction).

C) Lesion adjacent normal overall immune cell proportion of total cell comparison between 129S4 K (green color) and 129S4 U (orange color) models at matched stages. ((* p<0.05, ** p<0.01, and *** p<0.001, Two-tailed student's test).

D,E) Lesion adjacent normal immune cell subtypes proportion of total cell across five histological stages of 129S4 K (green color) and 129S4 U (orange color) models. (Pairwise multiple Welch's t-tests with Bonferroni correction).

F) Lesion adjacent normal immune cell subtypes proportion of total cell comparison between 129S4 K (green color) and 129S4 U (orange color) models at matched stages. ((* p<0.05, ** p<0.01, and *** p<0.001, Two-tailed student's test).

G,H) Lesion adjacent normal overall immune cell density across five histological stages of 129S4 K (green color) and 129S4 U (orange color) models. (Pairwise multiple Welch's t-tests with Bonferroni correction).

I) Lesion adjacent normal overall immune cell density comparision between 129S4 K (green color) and 129S4 U (orange color) models at matched stages. ((* p<0.05, ** p<0.01, and *** p<0.001, Two-tailed student's test).

J,K) Lesion adjacent normal immune cell subtypes density across five histological stages of 129S4 K (green color) and 129S4 U (orange color) models. (Pairwise multiple Welch's t-tests with Bonferroni correction).

L) Lesion adjacent normal immune cell subtypes density comparison between 129S4 K (green color) and 129S4 U (orange color) models at matched stages. ((* p<0.05, ** p<0.01, and *** p<0.001, Two-tailed student's test).

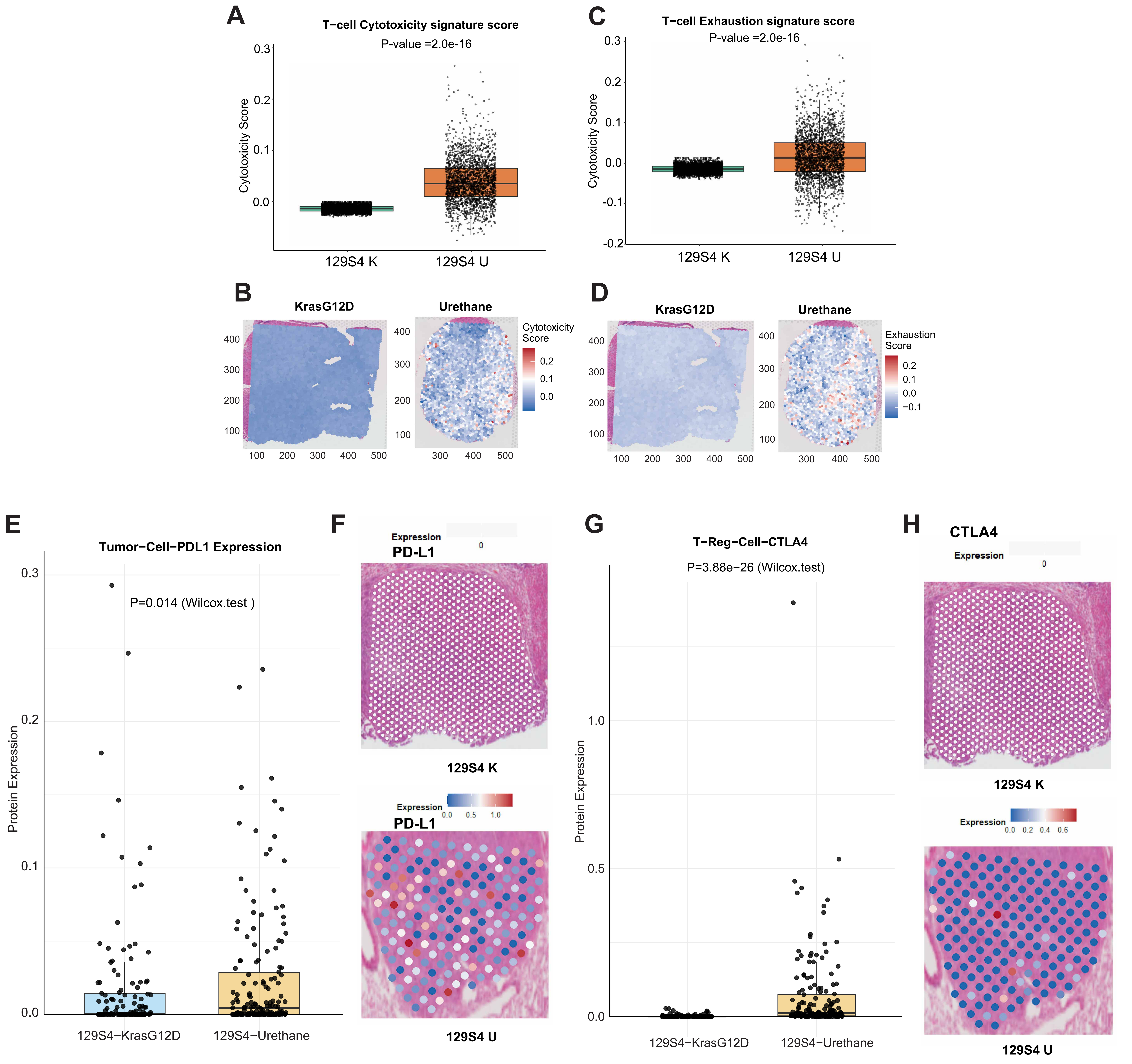

Fig. 8 | Cytotoxicity and exhaustion analysis on 129S4 KrasG12D and 129S4 Urethane models.
A) T cell cytotoxicity signature score between 1294 U and 129SK model (Wilcox test for P value).
B) Representative ST images showing the cytotoxicity signature score of 1294 U and 129SK model.
C) T cell exhaustion signature score between 1294 U and 129SK model (Wilcox test for P value).
D) Representative ST images showing the exhaustion signature score 1294 U and 129SK model.
E) Tumor cell PD-L1 expression on IMC data (Wilcox test for P value)
F) Representative ST images showing the PD-L1 expression of 1294 U and 129SK model.
G) Treg cell CTLA4 expression on IMC data (Wilcox test for P value).
H) Representative ST images showing the CTLA4 expression of 1294 U and 129SK model.

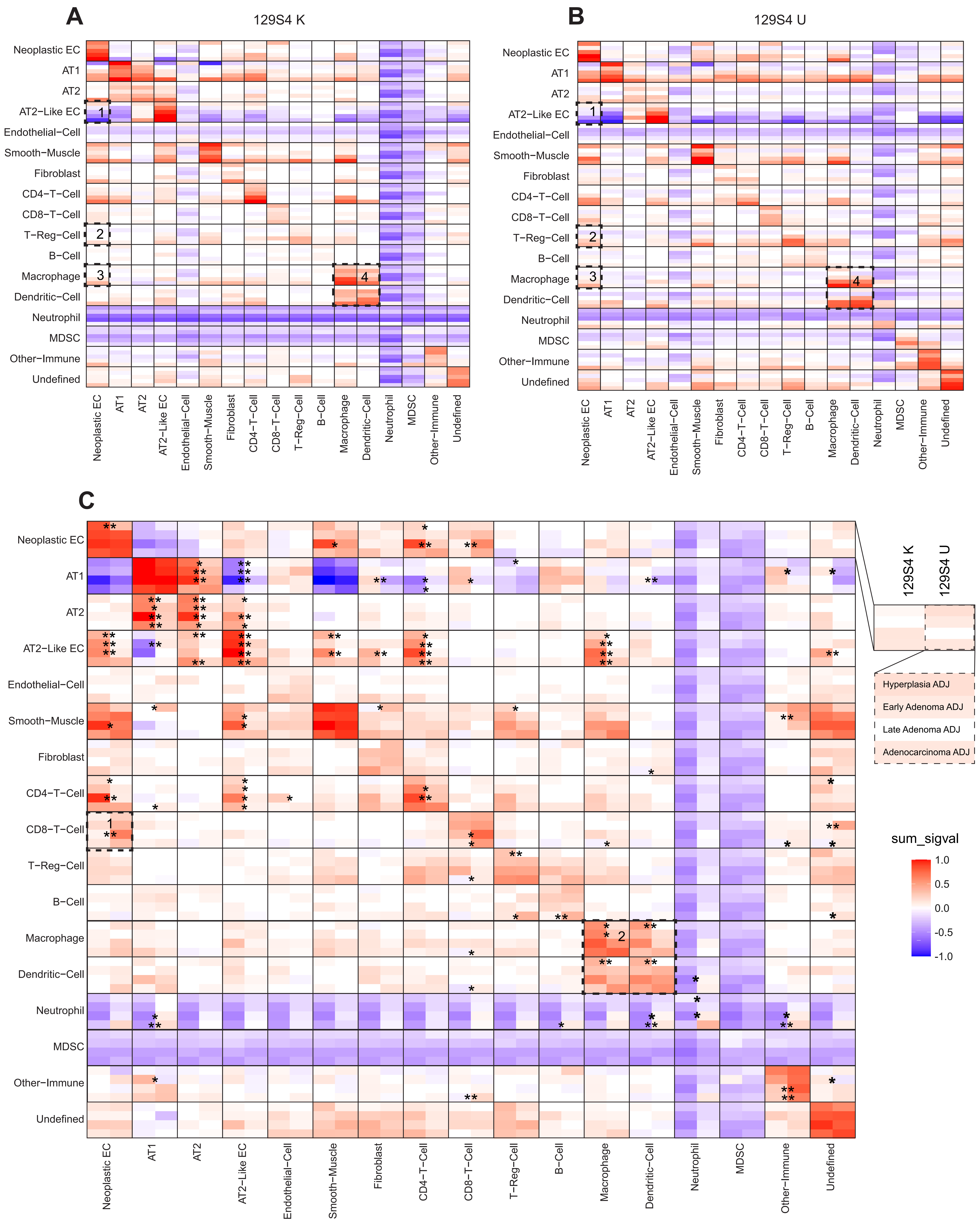

Extended Data Fig. 9 | Lesion inside and adjacent normal paired cell-cell interaction of 129S4 K and 129S4 U models

A) Heat map depicting significant pairwise cell–cell interaction (red) or avoidance (blue) across the five histological lesion (ROIs = 126) inside area of 129S4 K model. The black boxes depict associations referenced in the text.

B) Heat map depicting significant pairwise cell–cell interaction (red) or avoidance (blue) across the five histological lesion (ROIs = 158) inside area of 129S4 U model. The black boxes depict associations referenced in the text.

C) Heat map depicting significant pairwise cell–cell interaction (red) or avoidance (blue) comparison between 129S4 K and 129S4 U models across the five pathological stages (Normal, Hyperplasia, E-Adenoma, L-Adenoma, Adenocarcinoma) at lesion adjacent normal area. The black boxes depict associations referenced in the text. ((* $p<0.05$, ** $p<0.01$, and *** $p<0.001$, Two-tailed student's test)

A

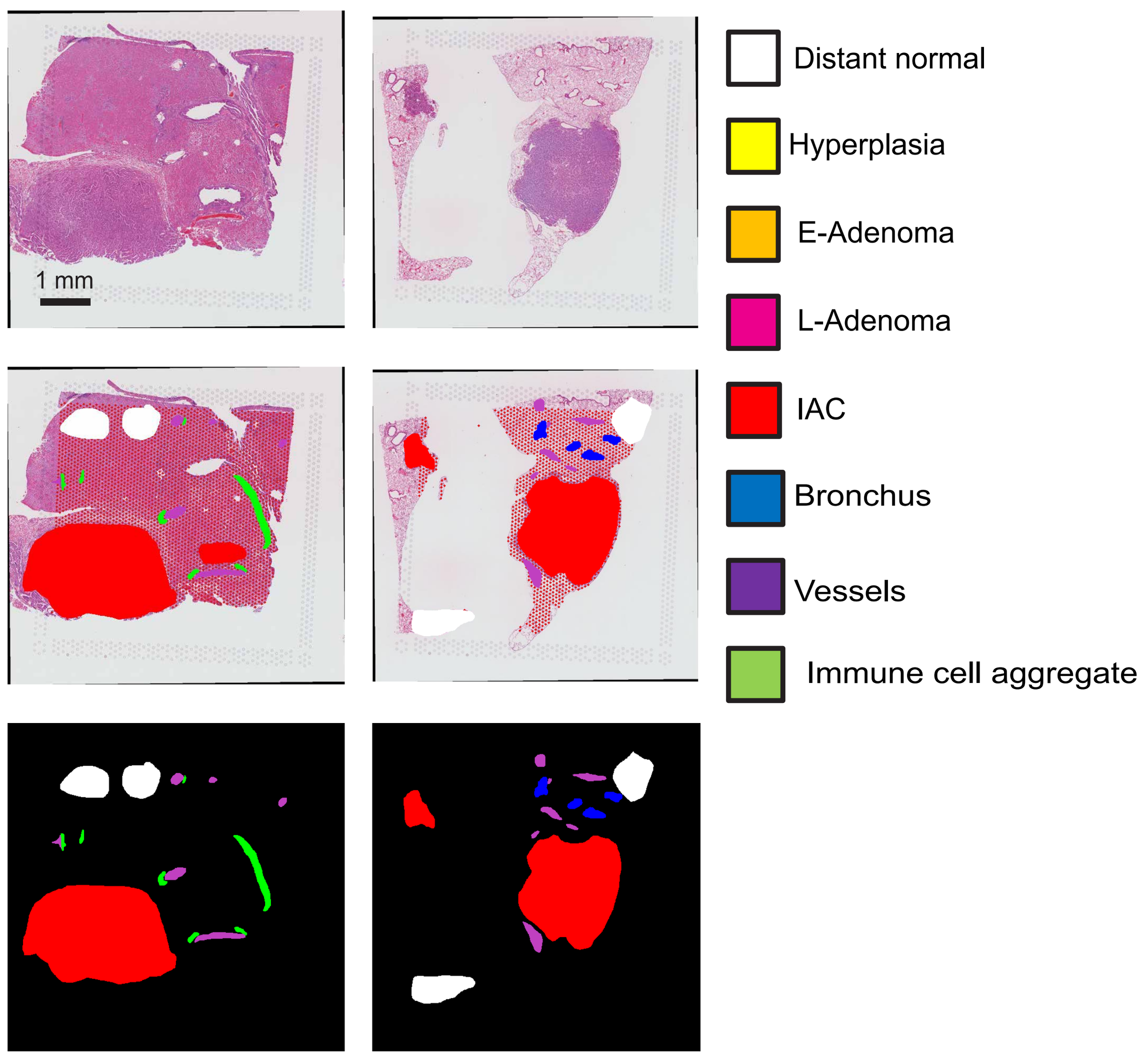

Extended Data Fig. 10 | 129S4 KrasG12D and 129S4 Urethane models spatial transcriptomics analysis
A) Pathological annotation of ST H&E images and spots mapping of 129S4 K and 129S4 U models, each color represents one type of ST spots area. Scale bars, 1 mm.
