# Supplementary material for: Spatial Profiling Reveals Distinct Molecular and Immune Evolution of Mouse Lung Adenocarcinoma Precancers with or Without Carcinogen Exposure
Source: Adv Sci (Weinh). 2026 Jan 25;13(17):e12597. doi: 10.1002/advs.202512597 (PMC13042775; doi:10.1002/advs.202512597)
Supplement: Supplementary file 2 — Supporting File 2: advs73897‐sup‐0002‐TableS1‐S7.zip. [file ADVS-13-e12597-s001.zip › Supplementary Table 1.pdf]

Metal	Marker	Clone	Catalog#	Dilution	Vendor
89Y	dsDNA	35I9 DNA	ab27156	500	Abcam
113In	Vimentin	EPR3776	ab193555	400	Abcam
115In	CD31	EPR17259	ab225883	400	Abcam
141Pr	aSMA	EPR5368	ab220795	400	Abcam
142Nd	Pan-CK	AE1/AE3	ab80826	200	Abcam
143Nd	CD44	EPR18668	ab232556	200	Abcam
144Nd	CD11c	EPR21826	ab240558	300	Abcam
145Nd	ECAD	4A2	ab233766	500	Abcam
146Nd	Beta-tublin	EPR16774	ab232361	600	Abcam
147Sm	CD326	EPR20533-63	ab228876	200	Abcam
148Nd	ICOS	EPR20560	ab225577	100	Abcam
149Sm	CCR2	EPR20844-15	ab273061	200	Abcam
150Nd	PD-L1	D5V3B	64988BF	50	CST
151Eu	CD49b	EPR5788	ab271894	100	Abcam
152Sm	CTLA4	CAL49	ab251599	200	Abcam
153Eu	CD14	EPR3653	3153025D	100	Standard BioTools
154Sm	TIM-3	EPR22241	ab242080	200	Abcam
155Gd	SPC	EPR19839	ab222929	500	Abcam
156Gd	F4/80	SP115	ab240946	100	Abcam
157Gd	Granzyme-B	EPR22645-206	ab255868	100	Abcam
158Gd	CD25	EPR22588-18	ab255858	100	Abcam
159Tb	CD4	CAL4	ab251608	200	Abcam
160Gd	iNOS	SP126	ab239990	200	Abcam
161Dy	NKP46	EPR23097-35	ab267792	100	Abcam
162Dy	CD8a	CAL38	ab251609	200	Abcam
163Dy	Ly-6G	EPR22909-135	ab261916	100	Abcam
164Dy	Arginase	EPR22033-369	ab259271	100	Abcam
165Ho	PAX5	D7H5X	716502-100	100	Ionpath
166Er	TTF-1	EPR5955(2)	ab227574	400	Abcam
167Er	Foxp3	EPR22102-37	ab244242	100	Abcam
168Er	CD127	EPR2955(2)	3168026D	100	Standard BioTools
169Tm	RAGE	EPR21171	ab228861	300	Abcam
170Er	CD3e	SP162	ab245731	150	Abcam
171Yb	CD11b	EPR1344	ab216445	200	Abcam
172Yb	Ki-67	SP6	ab197547	400	Abcam
173Yb	CD21	EP3093	ab271855	100	Abcam
174Yb	CD206	E6T5J	717404-100	100	Ionpath
175Lu	CD45	EPR20033	ab229292	200	Abcam
176Yb	Na-K-ATPase	EP1845Y	ab167390	500	Abcam
191&193Yb	Iridium		201192A	200	Standard BioTools
