# Supplementary material for: Spatial Profiling Reveals Distinct Molecular and Immune Evolution of Mouse Lung Adenocarcinoma Precancers with or Without Carcinogen Exposure
Source: Adv Sci (Weinh). 2026 Jan 25;13(17):e12597. doi: 10.1002/advs.202512597 (PMC13042775; doi:10.1002/advs.202512597)
Supplement: Supplementary file 2 — Supporting File 2: advs73897‐sup‐0002‐TableS1‐S7.zip. [file ADVS-13-e12597-s001.zip › Supplementary Table 2.pdf]

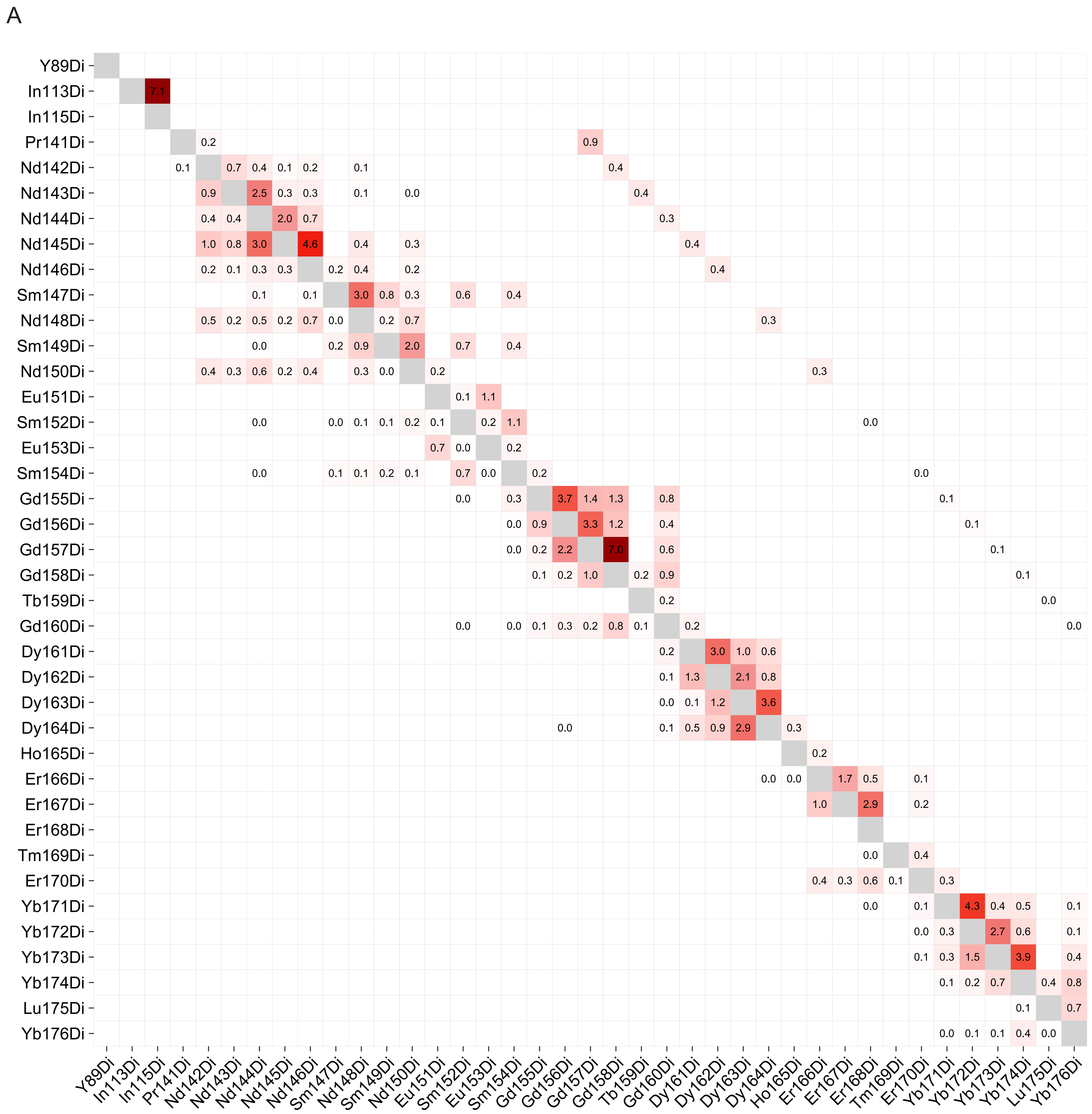

Figure S4. Spillover calculation

A, Spillover matrix calculated based on single-stained beads. Values on the diagonals are 1. By default, spillover is calculated only in potentially affected channels, which include M±1, those corresponding to known isotopic impurities, and M+16 (Figure S2D). Numbers in the cells indicate percentages of spillover by channels in rows into channels in columns. Numbers in the last column show the total amount of signal received in the corresponding channels.
